# Targeting Peptidergic Systems for Melanoma Treatment

**DOI:** 10.3390/cancers18091347

**Published:** 2026-04-23

**Authors:** Manuel L. Sánchez, Riffat Mehboob, Rafael Coveñas

**Affiliations:** 1 Laboratory of Neuroanatomy of the Peptidergic Systems, Institute of Neurosciences of Castilla and León (INCYL), University of Salamanca, c/ Pintor Fernando Gallego 1, 37007 Salamanca, Spain; lisardosanchez8@gmail.com; 2 Research and Development Department, Lahore Medical Research Center, Lahore 54000, Pakistan; mehboob.riffat@gmail.com; 3 Rotogen Biotech, Lahore 54000, Pakistan; 4 Group GIR USAL: BMD (Bases Moleculares del Desarrollo), University of Salamanca, 37007 Salamanca, Spain

**Keywords:** melanoma, peptides, anticancer peptides, oncogenic peptides, peptidergic systems, peptide receptor antagonists

## Abstract

Melanoma is a heterogeneous, complex and aggressive disease that, despite recent advances in molecular-targeted drugs and molecular and genetic analysis, represents approximately 65% of skin cancer deaths, and unfortunately survival dramatically decreases in melanoma stages III/IV. In young people there is an increased incidence of developing melanoma; hence new therapeutic strategies must be urgently investigated. Peptidergic systems play a crucial role in these strategies to fight melanoma. The scope of this review is to show the enormous potential of targeting peptidergic systems alone or in combination therapy with standard therapeutic strategies currently used in clinical practice to treat melanoma. In this sense, key points such as peptidergic systems and anti-melanoma treatments, oncogenic/anti-melanoma peptides, peptide receptors, peptidergic systems, melanoma risk and immune system relationships, clinical relevance, peptidergic systems and delivery strategies in melanoma will be discussed. Peptides exert oncogenic, anti-melanoma and dual oncogenic and anti-melanoma effects in melanoma, showing a high functional complexity in regulating melanoma development. A plethora of anti-melanoma strategies have been developed or repurposed for potential clinical applications, including peptide/peptide receptor antibodies, peptide receptor antagonists or agonists, enzyme inhibitors, CAR-macrophages, microRNAs and vaccines. Strategies for peptide delivery and protection from enzymatic degradation have also been developed. Some of the previous anti-melanoma strategies are based on the expression/overexpression of peptide receptors in melanoma cells which is crucial for diagnosis, melanoma risk and progression and metastasis development and for the application of more specific and safer anti-melanoma strategies. A meticulous and in-depth study of the peptidergic systems may help to understand how peptidergic systems regulate melanoma progression and shed light on possible therapeutic applications that can be applied in clinical practice. This review shows the enormous potential of targeting peptidergic systems alone or in combination therapy with standard therapeutic strategies currently used in clinical practice to treat melanoma. The benefits to be gained from these studies will be enormous because the peptidergic systems are promising antitumor targets in melanoma, based on the numerous anti-melanoma strategies that have been developed until now.

## Simple Summary

The peptidergic systems exert oncogenic, anti-melanoma and dual oncogenic and anti-melanoma effects in melanoma. A plethora of anti-melanoma strategies have been developed or repurposed for potential clinical applications. Anti-melanoma strategies are based on the expression/overexpression of peptide receptors in melanoma cells which is crucial for diagnosis, melanoma risk and progression and metastasis development and for the application of more specific and safer anti-melanoma strategies. This review shows the enormous potential of targeting peptidergic systems alone or in combination therapy with standard therapeutic strategies to fight melanoma.

## 1. Introduction

The heterogeneous, complex and aggressive tumor named melanoma arises from melanocytes; it exhibits a high metastatic potential and, in many cases, unfortunately does not respond to radiotherapy, chemotherapy or immunotherapy. Melanoma shows the highest mortality rate among all skin cancer types. Furthermore, when it spreads from the primary site to distant locations, few therapeutic options remain available [1]. Survival dramatically decreases in melanoma stages III/IV. Melanoma represents approximately 65% of skin cancer deaths and 1% of all cancers [2]. Moreover, in young people there is an increased incidence of developing melanoma [3]. Most melanomas are associated with exposure to ultraviolet radiation from sunlight, and a review on this radiation and melanomagenesis has been published [4], but melanomas (15%) also occurred in patients with a family history [4]. In fact, CDKN2A mutations are accountable for most of the hereditary melanomas, although other susceptibility genes have been reported (e.g., *BAP1*, *melanocortin 1 receptor*, *POT1*, *ACD*, and *TERT*) [5]. The histopathologic classification and prognostic factors of melanoma have also been reported [6] and the National Comprehensive Cancer Network (NCCN) guidelines for cutaneous melanoma indicated recommendations for the treatment, staging, and diagnosis of patients suffering from melanoma [7]. Moreover, the tumor-specific antigens expressed in melanoma cells pave the way for the development of melanoma immunotherapy [8]. This is the case of the immunogenic melanoma-overexpressed antigen (meloe) peptide which is overexpressed in melanoma cells compared with healthy melanocytes [9]. The meloe-1 antigen is involved in adoptive T cell transfer efficiency; hence this antigen is an important therapeutic target for melanoma immunotherapy [9,10]. However, despite recent advances in molecular and genetic analysis; proteomic, transcriptomic and genomic technologies; programmed cell death-1 protein and BRAF-V600E blockers (e.g., treatment with immune checkpoint inhibitors is associated with an exacerbation of autoimmune-related diseases); as well as molecular-targeted drugs which improved melanoma prognosis, melanoma cells can develop resistance to targeted strategies and become resistant [11,12,13,14]. This means that new therapeutic strategies must be urgently investigated to fight melanoma in combination with standard strategies such as surgery, chemotherapy, radiotherapy and immunotherapy. Thus, new therapies, but also better surveillance and prevention and earlier detection of melanoma, will help to decrease the mortality rate [11].

As occurs in other cancers (e.g., breast cancer) [15], peptidergic systems play important roles in the development of melanoma [3]. A review published in 2021 was focused on the involvement of neurotransmitters (e.g., catecholamines, serotonin, and glutamate), neurohormones (e.g., corticotropin-releasing hormone, α-melanocyte-stimulating hormone, and thyrotropin-releasing hormone) and peptides (e.g., substance P, galanin, calcitonin gene-related peptide, bradykinin, neuropeptide Y, and enkephalins) in melanoma [3]. A meticulous and in-depth study of the peptidergic systems may help to understand how they regulate melanoma progression and shed light on possible clinically applicable therapeutic strategies. Therefore, the aims of this review are to update the knowledge on the peptides published in the aforementioned review (e.g., substance P, neuropeptide Y, bradykinin, vasoactive intestinal peptide, and gastrin-releasing peptide), as well as reviewing other peptides not mentioned there (e.g., adrenomedullin, angiotensin, endothelin, kisspeptin, melittin, and neurotensin); to understand how oncogenic and anti-melanoma peptides regulate this disease; based on the existing data, to develop therapeutic strategies and select compounds with anti-melanoma activity for future investigation; and finally, to suggest future research lines on melanoma. In summary, this review highlights the roles played by the peptidergic systems in melanoma and shows the great therapeutic potential of these systems in the treatment of melanoma.

## 2. Peptidergic Systems and Melanoma

In this section the oncogenic and anti-melanoma actions of the numerous peptidergic systems that regulate melanoma development will be discussed.

### 2.1. Adrenomedullin

The release of adrenomedullin from melanoma cells favors tumor growth via lymphangiogenesis and angiogenesis [16]. This study was performed in MeWo, SK-MEL-28 and A375 melanoma cells expressing adrenomedullin and its receptor. Adrenomedullin expression was favored under hypoxic conditions, and the proliferation of SK-MEL-28 and A375 cells was decreased with anti-adrenomedullin or anti-adrenomedullin receptor antibodies [16]. Moreover, adrenomedullin augmented the migration/invasion of tumor cells and both processes were decreased with anti-adrenomedullin receptor antibodies; these antibodies also blocked lymphangiogenesis and angiogenesis and reduced the proliferation of MeWo xenografts leading to tumor regression [16]. Thus, adrenomedullin acted as an autocrine/paracrine agent favoring the proliferation, migration and invasion of melanoma cells and, in addition, promoted neovascularization and tumor growth favoring signals for both lymphangiogenesis and neoangiogenesis.

Another study has shown that an mRNA vaccine directed against the fusion protein of keyhole limpet hemocyanin-mouse adrenomedullin decreased angiogenesis and tumor burden in an animal experimental model (syngeneic metastatic melanoma model: C57BL/6 mice, B16F10 melanoma cells were administered to promote lung metastases) [17]. Thus, the size and number of lung metastases as well as the number of blood vessels were decreased in the adrenomedullin-immunized animal group; moreover, in this group the number of CD8^+^ T cells was higher than in the control group [17]. Antibody titers against adrenomedullin were also higher in immunized mice than in control animals. The same research group has recently reported that a stable mRNA vaccine against adrenomedullin decreased tumor burden and angiogenesis in a subcutaneous melanoma model (C57BL/6 mice, B16F10 melanoma cells administered) without inducing an immunosuppressive tumor microenvironment [18]. The immunization increased the number of CD8^+^ T cells and the anti-adrenomedullin IgG titers; a delay in tumor initiation was observed; and the tumor volume/area occupied by tumor blood vessels was reduced; however, weight loss and systemic toxicity signs were not observed, nor was the impairment of Ki67^+^ melanoma cell proliferation or changes in the tumor infiltration of Arg1^+^, FoxP3^+^, CD8^+^ and CD4^+^ cells reported [18]. In sum, this vaccine blocked angiogenesis and tumor initiation was delayed without favoring an immunosuppressive tumor microenvironment.

### 2.2. Angiotensin

The presence of components of the renin–angiotensin system (RAS, e.g., angiotensin II type 2 receptor, angiotensin-converting enzyme) has been observed in cancer stem cells in neck and head metastatic malignant melanoma [19]. The angiotensin-converting enzyme was overexpressed in melanoma cells, compared with melanocytes (Melan-a), and the activation of the angiotensin I-converting enzyme (ACE, converts angiotensin I to angiotensin II) by angiotensin II (ACE also acts as a receptor for this peptide) promoted TM-5 melanoma cell proliferation (murine, express ACE but not angiotensin type 1/2 receptors) [20]. Melanoma cell proliferation was inhibited with ACE silencing or ACE inhibitors (lisinopril), and angiotensin II did not affect the proliferation of melanocytes [20]. Angiotensin II also decreased the expression of the focal adhesion structural protein vinculin in TM-5 cells suggesting that this decrease favored the detachment and migration of melanoma cells [20]. In a study performed in human MV3 melanoma cells, lisinopril promoted cell invasiveness by favoring matrix metalloproteinase-2 secretion, and EMA401 (angiotensin II type 2 receptor antagonist) and losartan (angiotensin II type 1 receptor antagonist) also promoted the migration and invasion of these melanoma cells [21]. Another study has shown that angiotensin II increased Na^+^/H^+^ exchanger isoform 1 activity and decreased the migratory activity of human MV3 melanoma cells expressing both angiotensin II type 1/2 receptors and that losartan blocked cell migration and Na^+^/H^+^ exchanger isoform 1 activity in MV3 melanoma cells but favored the adhesion and invasion of these cells [22]. In this study, PD-123,319 (angiotensin II type 2 receptor antagonist) did not affect cell proliferation and migration or Na^+^/H^+^ exchanger isoform 1 activity but increased melanoma cell adhesion and invasion [22]. These findings are important since angiotensin II type 1 receptor antagonists favored MV3 melanoma cell migration, but angiotensin II type 2 receptor antagonists did not affect the migration of the same cells [21,22], and this means that angiotensin receptors mediate different mechanisms in melanoma cells. Furthermore, this could explain the contradictory results sometimes observed in published works because the different receptor types expressed and mediating different actions were not fully studied in melanoma cells.

Angiotensin II favored the lung metastasis of melanoma via the activation of adhesion molecules in vascular endothelial cells; the study was performed in C57BL/6 mice injected intravenously with B16F10 melanoma cells (these cells do not express angiotensin II type 1 receptors) [23]. The number of metastatic colonies was higher in animals treated with angiotensin than that observed in control mice. Valsartan (angiotensin II type 1 receptor antagonist) blocked the effect of angiotensin II; this peptide augmented the mRNA expression of E-selectin in vascular endothelial cells obtained from lung tissues and favored melanoma cell adherence to the vascular endothelium, and anti-E-selectin antibodies inhibited lung metastases induced by angiotensin II [23]. In sum, angiotensin favored hematogenous lung metastases by promoting the adhesion of B16F10 melanoma cells (mediated by E-selectin) to vascular endothelial cells. Angiotensin II/Y6AII (angiotensin II type 2 receptor agonist) promoted the proliferation of human melanoma cells (PMWK, SK-MEL-23, SK-MEL-224) expressing angiotensin II type 2 receptors, and EMA401/PD-123,319 (angiotensin II type 2 receptor antagonists) blocked angiogenesis and melanoma growth and potentiated MEK/BRAF inhibitors in cells with V600 mutations [24]. Moreover, ectopic AGTR1 expression (angiotensin II type 1 receptor is encoded by AGTR1) in melanoma cell lines missing endogenous expression blocked cell proliferation; hence AGTR1 exerted a suppressor role in melanoma [24]. Thus, depending on the angiotensin II type receptor, angiotensin II exerted an oncogenic or an anticancer action in melanoma [24].

Melanoma metastasis is mediated by the caveolin-1-Rab5 (Ras-related protein 5A)–Rac-1 (Ras-related C3 botulinum toxin substrate) signaling pathway [25]. The activation of angiotensin II type 2 receptors expressed in B16F10 melanoma cells inhibited cell and transendothelial migrations as well as metastasis, and the silencing of this receptor restored the previously inhibited effects [25]. Moreover, the activation of angiotensin II type 2 receptors decreased the transendothelial migration of A375 melanoma cells expressing caveolin-1, decreased the activity of Rac1/Rab5 and caveolin-1 phosphorylation, increased the activity of non-receptor protein tyrosine phosphatase 1B, and decreased the lung metastases of B16F10 melanoma cells administered in C57BL/6 mice [25]. This study showed that angiotensin II type 2 receptors reduced tumor cell migration, invasion and metastasis by protein tyrosine phosphatase 1B-mediated caveolin-1 dephosphorylation and the blockade of the CAV1-Rab5-Rac-1 signaling pathway [25].

The administration of angiotensin receptor inhibitors in mice (C57BL/6) with transplanted malignant melanoma cells (B16) increased antigen-specific T-cell response in tumors, decreased regulatory T cells, increased tumor-infiltrating T cells, and decreased the level of CCL5 in blood [26]. The co-administration of anti-programmed death-1 antibodies and the angiotensin receptor antagonist valsartan exerted a higher antitumor growth blockade than that found with a monotherapy administration [26]. This study showed that the therapeutic efficacy of blocked anti-programmed death-1 antibodies was re-established by inhibiting the angiotensin system. This is important because by increasing the anticancer actions of these antibodies, tumor-induced immunosuppression can be reversed.

A meta-analysis and systematic review have recently studied the relationships between melanoma and antihypertensive treatments [27]. An increased risk and dose–response relationship between ACE inhibitors and melanoma has been reported. Resistance to melanoma treatments has been attributed to the occurrence of cancer stem cells (controlled by the immune system and renin–angiotensin system) in the tumor microenvironment and, in this sense, a review focused on the renin–angiotensin system and cancer stem cells in the melanoma tumor microenvironment has been recently published [28]. This review pointed out that the renin–angiotensin system, through pro-renin receptors, regulated the phosphoinositide 3-kinase (PI3K)–protein kinase B (Akt)–mechanistic target of rapamycin (mTOR) and Ras-Raf-mitogen-activated protein kinase (MAPK)–extracellular signal-regulated kinase (ERK) signaling pathways and that this could be associated with treatment resistance and cancer stem cells [28]. Thus, the efficacy of therapeutic strategies (e.g., immunotherapy and targeted therapies) could be improved by regulating the renin–angiotensin system.

### 2.3. Bradykinin

The effects of bradykinin, mediated by kinin receptors, on melanoma are contradictory [29]. The activation of kinin B_1_ receptors counteracted melanoma tumor growth and metastasis [30]. In this study, the TM-5 melanoma cell line (expresses kinin B_1_ receptors but not kinin B_2_ receptors) was studied. In these cells, the activation of kinin B_1_ receptors decreased the formation of tumors in vivo and blocked the migration of melanoma cells in vitro; the administration of the kinin B_1_ receptor agonist des-Arg^9^-bradykinin (DABK) to melanoma tumors developed in C57BL/6 mice reduced the presence of immune cells in the tumor region, augmented the level of pro-inflammatory cytokines involved in the antitumor immune response, and reduced the number of mitotic cells, and a poorer vascular network and no invasion of surrounding tissues/metastasis were observed and increased survival was reported [30]. Thus, aggressiveness was considerably reduced; hence the kinin B_1_ receptor exerted a protective role against melanoma progression. As previously, another study confirmed that this receptor plays an important protective role in melanoma progression in mice [31]. B16F10 melanoma cells, derived from C57BL/6 mice, were administered to wild-type C57BL/6 and kinin B1 receptor knockout mice. The latter animals, compared with wild-type mice, showed an increased mitotic index, a higher activation of the Akt/ERK1/2 proliferative pathways, higher interleukin-10 levels, higher number of lung metastatic colonies, increased ulceration incidence, and lower CD8^+^ immune cells [31]. These findings suggest that kinin B1 receptor knockout mice showed a more aggressive metastatic onset and hence a worse prognosis, and showed that the kinin B1 receptor is involved in melanoma progression. Another study has confirmed the findings reported above because the activation of the kinin B1 receptor by DABK decreased melanoma metastasis in mice [32]. This treatment, compared with control animals, augmented CD8^+^ T-cell recruitment in the metastatic region favoring the host immune response, decreased vascular cell adhesion molecule 1 expression and, by targeting tumor cells, reduced the number of lung metastatic colonies [32]. In sum, DABK exerted a dual action: fighting against melanoma cells and favoring the immune reaction. However, a recent study has reported that bradykinin favored the migration and invasion of murine melanoma cells (Nex10C and Nex8H cells which are sub clones of the murine melanoma B16F10-Nex2 cell line) via the synthesis of nitric oxide and superoxide [33]. Nex10C has the capacity to colonize the lung and Nex8H is a highly invasive melanoma cell line. Nitric oxide promoted the migration/invasion of melanoma cells via the signaling axis involving PI3K, Rac1 and Ras [33].

The µ-opioid receptor agonist fentanyl citrate decreased the release of endothelin-1, which contributes to melanoma progression, mediated by bradykinin in mouse B16-BL6 melanoma cells [34]. This effect was attenuated with the µ-opioid receptor antagonist naloxone methiodide. This study also reported that B16-BL6 melanoma cells expressed µ-opioid, endothelin-1 and kinin B_2_ receptors.

### 2.4. Calcitonin Gene-Related Peptide

Calcitonin gene-related peptide promoted apoptosis in B16F10 melanoma cells, increased the expression levels of total/cleaved caspases 3/9, downregulated Bcl-2 expression, upregulated Bax expression, and increased the Bax/Bcl-2 ratio [35].

### 2.5. Corticotropin-Releasing Hormone

Corticotropin-releasing hormone (CRH) expression was studied in squamous cell carcinomas, basal cell carcinomas, melanocytic nevi and melanomas [36]. CRH decreased A431 melanoma cell proliferation; squamous cell carcinomas/basal cell carcinomas showed a lower CRH expression than that observed in melanomas; CRH expression was lower in melanocytic nevi than in primary melanomas; squamous cell carcinomas showed a lower CRH expression than basal cell carcinomas; and in metastatic melanomas, a lower CRH expression was observed in men than in women, and in men, a higher expression was associated with decreased overall survival [36]. Melanoma cell lines (DX-3 and G-361) expressed CRH; a high expression of both adrenocorticotropin and α-melanocyte-stimulating hormone was observed in malignant melanomas [37]; 7/12 cases of metastatic melanomas and 7/9 cases of primary melanomas showed the colocalization of pro-opiomelanocortin peptides and CRH; and CRH favored the expression of pro-opiomelanocortin mRNA in vitro which was abolished with the CRH antagonists α-helical CRH9-41 [38]. These studies indicate that the CRH–pro-opiomelanocortin axis is related to malignant melanomas. Moreover, CRH promoted the migration of melanoma cells via the ERK1/2 signaling pathway (CRH promoted ERK1/2 phosphorylation); this effect was blocked with the ERK1/2 blocker PD-098059 [3,39].

### 2.6. β-Endorphin

β-Endorphin expression was higher in advanced/metastatic melanomas than that observed in benign melanocytic nevi [3,40]. µ-Opioid-receptor-deficient (MOR^−/−^) and wild-type mice were administered with B16 melanoma cells, and human melanoma samples were studied for the expression of β-endorphin [41]. Opioids placed in the supernatant of B16 cells decreased the proliferation of normal but not MOR^−/−^ leucocytes [41]. B16 melanoma cells (producing β-endorphin) reduced tumor growth and increased the infiltration of immune cells inside the tumors in µ-opioid-receptor-deficient mice when compared with that observed in wild-type animals, and a positive association was reported between tumor progression and β-endorphin expression in human melanoma tissues [41].

### 2.7. Endothelin

Endothelin-1 contributes to melanoma cell proliferation, migration and invasion, and it is known that annexin A2 binds to endothelin receptors [42]. The phosphorylation of annexin A2 is needed for cell proliferation and Akt activation in endothelin-1 stimulated melanoma cells (SK-MEL28) [42]. Endothelin signaling favored melanoma tumorigenesis determined by constitutively active GNAQ [43]. Endothelin B receptors stimulated Gα_1_ and Gα_q_ proteins (downstream heterotrimeric G proteins), and constitutively active oncogenic versions of Gα_11_/Gα_q_ drove melanomagenesis [43]. Endothelin B receptors activated normal Gα_11_/Gα_q_ proteins even in presence of oncogenic Gα_q_ and it seems that tumorigenesis appeared to occur by activating the phospholipase C-beta (a Gα_q/11_ intermediate effector) [43]. This opens the possibility to target upstream receptors to counterbalance the actions mediated by hyperactive G proteins. An interplay between melanoma and endothelial cells and hypoxia controls cell motility and vascularization via vascular endothelial growth factor and endothelin-1 occurs [44]. This is important because tumor progression, aggressiveness and angiogenesis are determined by growth factor exchanges occurring between tumor and endothelial cells in the hypoxic tumor microenvironment [44]. In melanoma and endothelial lymphatic/blood cells, hypoxia increased endothelin-1 expression, favoring the release of vascular endothelial growth factors A and C, via the hypoxia-inducible growth factors 1α and 2α [44]. Vascular endothelial factors and endothelin-1 exerted autocrine/paracrine actions and favored morphological changes in endothelial cells located in lymphatic/blood vessels and aggressiveness, and conditioned media from endothelial cells increased vessel-like channel formation and melanoma cell migration [44]. This was inhibited with endothelin B receptor antagonists (A-192,621) or with vascular endothelial growth factor receptor 2/3 antibodies. Moreover, endothelin B receptor antagonists (A-192,621) decreased the number of lymphatic/blood vessels and reduced tumor growth in melanoma xenografts [44]. Another study demonstrated that the overexpression of endothelin-3 exerted an immunosuppressive effect in the melanoma microenvironment [45]. The study was performed in transgenic mice (K5-End3) overexpressing endothelin-3 in which melanoma cells were injected (YUMMER1.7, YUMM1.7, B16F10). Endothelin B receptors were expressed in immune cells; endothelin-3 favored the proliferation of regulatory T cells and FOXP3 expression in vitro; and melanoma tumors were sensitive to endothelin B receptor antagonists (BQ-788) and immune checkpoint inhibitors (anti-CTLA-4) [45]. Thus, endothelin B receptors mediated immunosuppressive effects, facilitating tumor immune escape and promoting melanoma progression.

The co-administration of endothelin B receptor antagonists and MAPK inhibitors is a promising treatment for patients with melanoma and hyperactivation of the MAPK signaling pathway [46]. This is important because, after treatment with MAPK inhibitors, most of the tumors develop resistance in melanoma patients with BRAF mutations. MAPK inhibitors exerted an antiproliferative action but increased the expression of endothelin B receptors, and when these receptors were activated by endothelin the proliferation of melanoma cells occurred [45]. Compared to the administration of MAPK inhibitors alone, the inhibition of both MAPK and endothelin/endothelin B receptor signaling pathways showed a higher anticancer effect, decreased tumor growth and increased survival [46]. Moreover, the endothelin/endothelin B receptor system did not promote resistance towards MAPK inhibitors by restoring its activity but through other signaling pathways downstream of the GNA_q/11_ proteins [46]. In sum, the blockade of the endothelin–endothelin B receptor system synergizes with MAPK inhibitors in BRAF-mutated melanomas. The combination of MAPK inhibitors with an antibody–drug conjugate targeting the endothelin B receptor has been tested in melanoma cell lines (A2058, SK-MEL-5, WM-266-4) and tumor models of melanoma [47]. In this study, the inhibition of MEK and/or BRAF (key proteins involved in cell proliferation and growth) augmented the endothelin B receptor expression in melanoma cells and increased the anticancer effect exerted by the antibody–drug conjugate, and melanoma cells acquired resistance to BRAF inhibition via the long-term cultured, retained drug-induced high expression of endothelin B receptors [47]. However, in normal melanocytes this expression was not enhanced when BRAF expression was inhibited. Thus, MAPK pathway blockade increased the efficacy of an anti-endothelin B receptor drug conjugate by favoring target expression in melanoma [47].

Another study has demonstrated that the monoclonal antibody rendomab B4 directed against the endothelin B receptor blocked the migration of melanoma cells (SLM8, WM-266-4, UACC-257) [48]. This receptor was overexpressed in these cells and played an important role in vascularization and tumor cell migration and proliferation; a previous antibody named rendomab B1, produced by DNA immunization, had antagonist properties against endothelin B receptors expressed in endothelial cells but showed a poor affinity for the endothelin B receptors expressed in melanoma cells [48]. This suggests a tumor-specific endothelin B receptor form. However, another antibody named rendomab B4 bound to endothelin B receptors expressed in melanoma cells (SLM8, WM-266-4, UACC-257) [48]. Rendomab B4, after binding to these receptors, was internalized and colocalized with EEA-1 (an endosomal protein) in UACC-257 melanoma cells; in addition, the antibody inhibited both cell migration and the phospholipase C pathway mediated by endothelin but failed to decrease ERK1/2 phosphorylation, also mediated by this peptide [48]. Thus, rendomab B4 blocked melanoma cell migration, and it is a melanoma-specific antibody for discriminating endothelin B receptors in these cells. A high-affinity single-domain antibody (nanobody) against endothelin B receptors has also been obtained, opening the possibility of developing new lines of research and clinical applications to treat melanoma [49]. A chimeric antibody–drug conjugated to monomethyl auristatin E named xiRB49-NMAE has also been developed, and it has been demonstrated that it exerts an antitumor effect against both melanoma cells and endothelin B receptor xenograft tumor models [50]. Moreover, after using immunohistochemical techniques, all the lymph node biopsies studied belonging to melanoma patients were positive to rendomab B49 antibody (RB49, shows a high affinity for the activated conformation of the endothelin B receptor) and this means that RB49 could be used as a diagnostic tool in melanoma patients [50]. CAR-macrophages directed against melanoma cells expressing the endothelin B receptor have been recently generated [51]. This study showed that CAR-macrophages exerted a high anticancer activity against WM266 melanoma cells expressing a high number of endothelin B receptors, but no activity was observed against A375 melanoma cells which express a low number of these receptors [51]. Previous studies pave the way for treating endothelin B receptor-expressing melanoma tumors.

A correlated expression of plexin C1 (a melanoma suppressor) and the endothelin B receptor (mediates both suppressive and promoting actions for melanoma) has been reported in this tumor [52]. The study was performed in melanomas obtained from RFP/RET-transgenic mice (RET) and from endothelin B receptor heterozygously deleted RET mouse (RET-endothelin B receptor); melanoma develops spontaneously in both mouse types [52]. Plexin C1 expression decreased in the latter animals compared with the expression found in the RET mice; plexin C1 transcript expression in melanomas from RET animals was higher than that observed in RET-endothelin B receptor mice, and a correlation between plexin C1 and endothelin B receptor expression was found in melanomas from both animal groups [52]. Moreover, a correlation between the protein and transcript expression levels of PLXNC1 and EDNRB has been reported in human primary melanomas, and PLXNC1 transcript expression levels were decreased in EDNRB-depleted human melanoma cells [52]. Plexin C1 expression decreased in parallel with endothelin B receptor expression, and plexin C1/PLXNC1 can be downstream of the endothelin B receptor/EDNRB signaling pathway; hence it can be associated with this pathway for the suppression of tumors [52]. The findings suggest that the positive correlated expression level of plexin C1/*PLXNC1* and the endothelin B receptor/*EDNRB* plays an important role in the endothelin B receptor/*EDNRB*-mediated melanoma suppressive action.

Melanoma and endothelial cells overexpressed endothelin-1 and endoglin (CD105, a transforming growth factor-β coreceptor) [53]. Accordingly, another anti-melanoma strategy has been the construction of a eukaryotic expression plasmid encoding the shRNA molecules against endoglin under the control of the endothelin-1 promoter. This strategy was tested in in vitro (SVEC4-10 endothelial cells and B16F10-luc melanoma cells) and in vivo (mouse with metastatic B16F10-luc tumor) experiments [53]. Plasmids showed anti-angiogenic and antiproliferative actions in endothelial cells and antimetastatic and anticancer effects in melanoma cells. Thus, targeting melanoma with shRNA molecules against endoglin is an effective anti-melanoma treatment. An attenuation of melanoma cells, by decreasing the nuclear factor kappa-light-chain-enhancer of activated B cells (NF-κB) and secreted protein acidic and rich in cysteine (SPARC) expressions, has been reported after the silencing of endothelin-3 [54]. In metastatic melanoma this peptide promoted melanoma cell survival, and it was expressed in cultured melanoma cells and metastatic melanoma tissues [54]. In this study, the endothelin-3 gene sequence-specific shRNA vector pLVTHM-endothelin-3-RNAi was obtained and transfected into A375 or MMRU melanoma cells. Compared with the control group, the transfection decreased melanoma cell proliferation, inhibited tumor growth and cell migration/invasion, increased apoptosis in tumor cells, and downregulated the expressions of SPARC and its upstream signaling molecule NF-κB [54]. Thus, the silencing of endothelin-3 suppressed the malignant behaviors of melanoma cells.

An endothelin-1 immunohistochemical study was performed in metastatic and invasive melanomas, melanoma in situ lesions, melanocytic nevi and blue nevi [55]. In the tumor microenvironment, human endothelin-1 expression increased with advancing stages of melanocyte transformation; hence this peptide plays a crucial role in melanoma progression and invasion [55]. Intratumoral administration of the endothelin B receptor antagonist BQ788 (3–10 mg) was performed in five patients suffering from melanoma [56]. No adverse effects were found; Ki67 and endothelin B receptor expressions decreased in three patients; an inverse relation between hypoxia-inducible growth factor 1α and the endothelin B receptor was reported in all patients studied, and the inhibition of tumor growth was observed in one patient (the only one treated for longer than one week, 10 mg) [56]. Although only a few patients were studied, this preliminary study is quite promising.

### 2.8. Galanin

Galanin has been reported in melanocytic nevi, cutaneous melanoma metastases and cutaneous melanomas [57]. In melanocytic nevi the expression of galanin was lower than that reported in melanomas, and an immunostaining positive correlation between galanin and α-melanocyte-stimulating hormone was observed in melanomas [57].

### 2.9. Gastrin-Releasing Peptide

A high expression of gastrin-releasing peptide receptors has been reported in cutaneous melanoma samples, but this expression was not related with pathological features associated with prognosis; in addition, no difference in receptor expression density was observed between metastatic or primary melanomas [58]. Gastrin-releasing peptide was observed in melanomas, and a high density of immunoreactivity for this peptide was found in nodular melanomas; moreover, gastrin-releasing peptide immunoreactivity was associated with an increase in the melanin pigment amount located in melanoma cells [59]. A study was performed in C57BL/6 mice administered with B16F10 melanoma cells and immunized with vaccines to create antibodies to block and destruct melanoma tumors [60]. Mouse granulocyte macrophage-colony stimulating factor (mGM-CSF) was fused with gonadotropin-releasing hormone (GnRH) and gastrin-releasing peptide (GRP) respectively to obtain mGM-CSF/mGGn (mGGn) and mGM-CSF/GRP6 (mG6) fusion proteins [60]. Anti-mGM-CSF/mGGn and anti-mGM-CSF/GRP6 vaccines inhibited melanoma tumors by decreasing tumor volume and weight; in addition, the combination of vaccines showed a more effective action than vaccines given separately [60].

### 2.10. Gonadotropin-Releasing Hormone

Melanoma cells expressed gonadotropin-releasing hormone (GnRH) receptors, and the activation of these receptors by GnRH agonists decreased the proliferation, migration, invasion and metastasis of melanoma cells [61]. In fact, the GnRH agonists zoladex (goserelin acetate) inhibited melanoma cell migration and invasion via the blockade of α-3 integrin/matrix metalloproteinase-2 activity and expression [61]. Thus, GnRH agonists exerted antiproliferative and antimetastatic effects against melanoma cells expressing GnRH receptors. Moreover, GnRH agonists (goserelin acetate) decreased melanoma angiogenesis by targeting melanoma cells and reduced the pro-angiogenic vascular endothelial growth factor expression in BLM melanoma cells as well as the expression and release of the vascular endothelial growth factor-165 isoform from these cells [62]. Thus, GnRH agonists exerted an anti-angiogenic action by reducing the release of vascular endothelial growth factor from melanoma cells.

### 2.11. Hemokinin-1

This peptide binds to the neurokinin-1 receptor [63]. Hemokinin-1 augmented the migration capacity of B16F10 and A375 melanoma cells; the neurokinin-1 receptor antagonist L-732,138 inhibited this effect and increased the expressions of matrix metalloproteinase-2 and membrane-type 1 matrix metalloproteinase in the melanoma cells [63]. Hemokinin-1 promoted ERK1/2, p38, and c-Jun N-terminal kinase phosphorylation by protein kinases A or B, and the activation of the kinases favored the expressions of membrane-type 1 matrix metalloproteinase and matrix metalloproteinase-2 and the migration of melanoma cells [63]. Thus, hemokinin-1 plays a crucial role in melanoma cell migration.

### 2.12. Kisspeptin

A study has shown the effects of daylight exposure on kisspeptin gene expression in the hypothalamus and melanoma tumor formation in mice [64]. New-born mice (BALB/c) were divided into daylight and blind (darkness) groups, and each group was subdivided into control and melanoma (B16F10 cells were administered). Kisspeptin hypothalamic expression was lower in healthy animals than in those with melanoma; thus, it seems that the tumor increased the synthesis of hypothalamic kisspeptins which exert anticancer (antiproliferative) and antimetastatic effects [64,65]. This hypothesis must be confirmed since another study showed a downregulation of kisspeptin mRNA expression in metastatic melanomas [66]. The blind group showed a lower hypothalamic expression of kisspeptin and tumor growth speed than that observed in the daylight group; kisspeptin synthesis was directly proportional to the duration of daylight exposure, and the tumor volume was directly proportional to the hypothalamic level of kisspeptin expression (tumor volume increased with increased hypothalamic kisspeptin expression) [64]. Thus, daylight exposure promoted a higher hypothalamic kisspeptin expression and tumor growth speed. Another study has demonstrated that the upregulation of the metastasis-suppressor gene KISS1 blocked melanoma cell (SK-MEL-3) proliferation and migration [65]. KISS1 and Let-7i (a microRNA) were downregulated in patients with melanoma; this microRNA decreased the expression of proliferation/metastasis-related genes in in vitro studies; Let-7i upregulation counteracted the proliferation and migration of SK-MEL-3 melanoma cells, and in addition, Let-7i promoted apoptosis in tumor cells [65]. Inhibitors of KISS1 favored melanoma cell proliferation and migration which were counteracted with Let-7i [65]. Moreover, kisspeptin 54 (derived from KISS1 cleavage) increased vemurafenib’s (zelboraf, a BRAF kinase inhibitor) pro-apoptotic effect in vemurafenib-resistant melanoma cells [67].

### 2.13. α-Melanocyte-Stimulating Hormone

Recent reviews have been published on the biology, anticancer strategies and clinical relevance of the α-melanocyte-stimulating hormone/melanocortin 1 receptor system in melanoma [68] and on the risks and benefits of chronic melanocortin 1 receptor activation [69]. The activation of this increased the DNA damage response of melanocytes to oxidative stressors and solar radiation [70]. The α-melanocyte-stimulating hormone/melanocortin 1 receptor system decreased melanoma risk development by maintaining melanocytes’ genomic stability by controlling the DNA damage response to solar ultraviolet radiation [71]. This study also showed that α-melanocyte-stimulating hormone and endothelin-1 interacted synergistically and favored melanogenesis and melanocyte cell proliferation and also blocked solar-ultraviolet-radiation-induced apoptosis [71]. This is important because DNA repair ability is essential for regulating melanoma risk. The ultraviolet B radiation or the exposure to α-melanocyte-stimulating hormone favored the melanoma cell synthesis (mouse Cloudman S91) of mRNAs for pro-opiomelanocortin and α-melanocyte-stimulating hormone, and this radiation promoted the synthesis and release of adrenocorticotropin and α-melanocyte-stimulating hormone from melanoma cells [72]. α-Melanocyte-stimulating hormone blocked the invasive and metastatic capacities of melanoma cells (B16-BL6) [3,73], reduced adhesiveness to laminin/fibronectin and partially inhibited the synthesis of matrix metalloproteinase 2/9 from melanoma cells [73]. Moreover, another study has shown that tumor necrosis factor-α favored the migration of melanoma cells (C8161, HBL) and that α-melanocyte-stimulating hormone inhibited the migratory effect induced by this factor in HBL cells but not in C8161 cells (showing a loss-of-function of the melanocortin 1 receptor) [74]. C8161 cell transfection with wild melanocortin 1 receptor produced melanoma cells whose migratory capacity was blocked with α-melanocyte-stimulating hormone [74]. Moreover, the migration of both previous melanoma cells was reduced with antibodies directed against the beta (1)-integrin subunit [74]. Another study confirmed previous findings [75]. α-Melanocyte-stimulating hormone exerted anti-invasive and anti-inflammatory effects in melanoma cells (HBL) expressing the wild-type melanocortin 1 receptor; melanoma cells (C8161, A375-SM) did not respond to α-melanocyte-stimulating hormone because both cell lines displayed melanocortin 1 receptor polymorphisms, and the invasion capacity of C8161 cells transfected with the wild-type melanocortin 1 receptor was blocked with α-melanocyte-stimulating hormone [75]. Cell adhesion molecules are involved in cellular cytotoxicity [76] and because cytotoxicity is associated with the expression level of these molecules in melanoma cells, a weak cell adhesion molecule expression could allow melanoma cells to escape from the surveillance of the immune system [76]. It is important to remark that the intercellular adhesion molecule 1 (ICAM-1) is less expressed in primary melanomas than in metastatic melanomas, and this differential expression could serve as a marker for the association between the clinical course of melanoma and lesion thickness [77]; it is also known that α-melanocyte-stimulating hormone blocked, via its receptor, the expression of ICAM-1-induced by the tumor necrosis factor-α in melanoma cells [78,79]. α-Melanocyte-stimulating hormone reduced the interaction between T lymphocytes and melanoma cells, favoring the escape of melanoma cells from the immune system [80]; blocked the activation of the inflammatory agent NK-κB in melanoma cells [81]; and decreased the invasion of uveal melanoma cells, through fibronectin, whereas tumor necrosis factor-α increased invasion [82], and the α-melanocyte-stimulating-hormone-dependent PI3K signaling pathway supported energy metabolism, through glucose uptake, influencing actin cytoskeleton and decreasing melanoma cell motility [83]. Pro-opiomelanocortin gene delivery blocked melanoma (B16F10 cells) metastasis and growth through an α-melanocyte-stimulating-hormone-induced blockade of the NK-κB-cyclooxigenase-2 signaling pathway; metastasis decrease was due to an attenuated adhesive and migratory capacity [84].

Human primary cutaneous melanoma showed a higher expression of α-melanocyte-stimulating hormone than that observed in melanocytic nevi; however, no expression was reported in melanoma metastases [85]. A high expression of α-melanocyte-stimulating hormone has been reported in malignant melanomas [37] and α-, β-, and γ-melanocyte-stimulating hormones have been observed in cutaneous malignant melanoma of nodular type [86]. Melanocortin 1 receptor expression has been suggested as a marker for melanoma progression [87]. Melanocyte-stimulating hormone receptors have been detected in melanoma cells but not in inflammatory tissues or adjacent connective tissues in human melanoma samples [88] and these receptors were not detected in all human samples studied (three patients showed a high density, five a low density, and three did not show receptors) [88]. Compared to cutaneous melanomas, the higher expression of the melanocortin 1 receptor was observed in primary and ulcerated lesions and mucosal melanomas, and this was related to shorter survival in metastatic and primary melanomas [87]. Moreover, another study has demonstrated that patients with melanoma showing a low expression of melanocortin 1 receptors had a better prognosis than those expressing a high level [89]. Thus, melanocortin 1 receptor expression could be a predictive factor for postoperative outcomes in patients suffering from melanomas. A high expression of melanocortin 1 receptor expression is related with impaired T cell infiltration; decreased CXCL9, 10, 11 expressions; and poor prognosis in patients with melanoma [90], and the activation of melanocortin 1 receptors in melanoma cells impaired tumor T cell infiltration, reducing anticancer immunity [90]. This is important because T cell infiltration blockade counteracts antitumor immunity and promotes resistance to immune checkpoint blockade therapies [90]. The loss of melanocortin 1 receptors in melanoma cells promoted the anticancer response of T cells and the resistance to the immune checkpoint blockade was overcome [90]. Melanocortin 1 receptor mediated the blockade of the interferon-γ-induced CXCL9, 10, 11 transcriptions leading to T cell infiltration impairment into the tumor microenvironment [90]. The anti-melanoma actions of ML00253764 (melanocortin 4 receptor antagonist) alone or in combination with vemurafenib (B-RafV600E inhibitor) have been studied in in vitro and in vivo experiments [91]. Human melanoma samples and cells (WN 266-4, A2058) expressed melanocortin 4 receptors, and when these receptors were blocked with ML00253764, pro-apoptotic and antiproliferative effects were observed via BCL-XL decrease and ERK1/2 phosphorylation blockade; the co-administration of vemurafenib and ML00253764 promoted a synergic action against melanoma cells, and, in addition, tumor growth was inhibited without promoting genotoxicity or weight loss [91].

It has been demonstrated that melanocortin 1 receptor variants are related to an increased cutaneous melanoma risk [92]. This receptor is overexpressed in melanoma cells (A375), and it can be targeted by ligand drug conjugates (by coupling the melanocortin 1 receptor agonist (e.g., melanotan-II) to cytotoxic drugs (e.g., camptothecin)); in fact, this ligand drug conjugate (melanotan-II-camptothecin) reduced the growth of A375 melanoma cells [13]. Similar strategies can be applied for melanoma imaging (in both cell cultures and solid tumors) using conjugates of melanocortin 1 receptor-targeting peptides and near-infrared fluorescent indocyanine dyes [93], and for the preparation of ^67^Ga-labeled NODAGA-conjugated lactam-cyclized α-melanocyte-stimulating hormone as a radiometal chelator for radiolabeling of α-melanocyte-stimulating hormone [94]. Other studies have reported similar strategies for melanoma diagnosis and treatment [95,96,97,98,99,100,101,102].

Finally, α-melanocyte-stimulating hormone increased citric, oxaloacetic, malic, and fumaric acid levels in B16F10 melanoma cells; this means that the peptide enhanced cellular energy metabolism [103], and 8-methoxybutin, a microphthalmia-associated transcription factor inhibitor, blocked α-melanocyte-stimulating-hormone-induced melanoma cell proliferation (B16F10) in in vitro and in vivo experiments by inhibiting the transactivation activity of this factor [104].

### 2.14. Melittin

A recent review has been published regarding the use of bee venom to treat skin cancer [105]. Bee venom and the peptide melittin (a major component of bee venom) blocked melanoma cell growth, migration, invasion and decreased the survival of melanoma cells (B16F10, SK-MEL-28, A375SM) by inhibiting the PI3K/Akt/mTOR and MAPK signaling pathways [106]. Bee venom and melittin promoted apoptosis in melanoma cells by increasing caspase 3 and 9 activities; favored the downregulation of the PI3K/Akt/mTOR and MAPK pathways, and the co-administration of temozolomide (a chemotherapeutic drug); and melittin increased the inhibition of melanoma cell growth and invasion compared to when melittin or temozolomide were administered alone [106]. Mitochondrial Ca^2+^ overload, through voltage-gated Ca^2+^ entry, favored the anti-melanoma action of diallyl trisulfide (DATS) [107]. DATS promoted apoptosis in melanoma cells (A375, A2058) in a Ca^2+^-dependent manner and favored mitochondrial Ca^2+^ overload via extracellular and intracellular Ca^2+^ fluxes, and the anti-melanoma effects of DATS were inhibited when Ca^2+^ channel blockers were administered [107]. Thus, DATS favored mitochondrial Ca^2+^ overload through a non-store-operated calcium entry, leading to an anti-melanoma effect. In the case of melittin, this peptide promoted a store-operated calcium entry and apoptosis [107]. Another study has demonstrated that melittin also exerted an anticancer effect against A375 melanoma cells (favoring apoptosis via the intrinsic mitochondrial pathway) [108]. Moreover, the peptide blocked the cell motility and migration of tumor cells by disrupting the actin cytoskeleton-epidermal growth factor receptor interaction and the epidermal growth factor receptor signaling pathway [108]. In fact, melittin blocked the expression of the epidermal growth factor receptor (a binding protein to F-actin).

M2-like tumor-associated macrophages are related to the invasiveness of melanoma cells and poor prognosis [1]. The synthetic peptide melittin–dKLA (a fusion of melittin with the pro-apoptotic peptide d(KLAKLAK)) favored apoptosis in M2-like tumor-associated macrophages; the blockade of M2 macrophage proliferation and migration by this synthetic peptide was associated in vivo with a decrease in melanoma tumor growth [109]. Melittin–dKLA induced a higher caspase 3 expression and cell death in M2 macrophages than in M0/M1 cells. Another study has shown similar results [1]. In this case, the administration of melittin–mertansine promoted apoptosis in M2 tumor-associated macrophages in mice (C57BL/6 animals administered with B16F10 melanoma cells) [1]. Mertansine is an antibody-conjugatable inhibitor of microtubules and, when conjugated with melittin, decreased M2-like tumor-associated macrophages leading to the blockade of melanoma cell growth, migration and invasion, improving the survival rate when compared with the administration of melittin or mertansine alone [1]. Melittin–mertansine also increased natural killer/CD8^+^ cytotoxic T cell infiltration in the tumor microenvironment [1]. Recently, a new strategy using multifunctional hepatitis B core virus-like particles (HBc VLPs) was developed to encapsulate the anticancer peptide melittin to treat subcutaneous melanoma and lung metastasis in mice [110]. This design incorporated M2pep (targeting M2 macrophages which exert an immunosuppressive effect), tuftsin (favoring phagocytosis), and RGD peptides (short amino acid sequences improving tumor specificity) [110]. HBc VLPs improved tumor selectivity, decreased cytotoxicity, protected melittin from enzymatic degradation and favored the suppression of tumors in both experimental models (subcutaneous melanoma and lung metastasis) [110]. In sum, this procedure was safe and effective for the delivery of melittin and melanoma treatment. Another study used bacteria instead of viruses to treat melanoma because the tumor microenvironment is favorable for the growth of anaerobes/facultative aerobes such as Salmonella [111]. *Salmonella typhimurium* was engineered to express and release RGD (Arg-Gly-Asp)–melittin, and then bacteria were administered to mice (BALB/c-nu) containing B16 melanoma xenografts [111]. The release of melittin promoted apoptosis and blocked melanoma cell proliferation, migration and invasion and also inhibited chemotaxis. The same study demonstrated that LH430/pRGD-MEL blocked tumor growth by activating phagocytosis and apoptosis, minimized host toxicity, disturbed the immune barrier inside the tumor, and increased the survival of animals [111]. Previous studies show the importance and high potential of microbial-mediated precision anti-melanoma strategies.

Hypochlorous-acid-treated tumor cells (B16F10) showed a strong stimulatory effect on macrophages and dendrite cells [112]. These cells were loaded into a melittin-encapsulated hydrogel scaffold and administered intratumorally; this procedure inhibited tumor growth, promoted tumor cell death and tumor-related macrophage reprogramming towards an M1 phenotype, favored cytotoxic T lymphocyte infiltration, increased the anticancer effects of the immune checkpoint blockade, and augmented the survival of mice with melanoma [112]. This study highlighted the importance of the delivery of anticancer agents and the generation of cell-derived secretions. The hybrid vaccine melittin-RADA32-CpG-lysate (MCL) killed melanoma cells and activated dendritic cells in vitro and favored the infiltration of cytotoxic T lymphocytes in the tumor microenvironment and the activation of dendritic cells in draining lymph nodes as well as exerted anticancer effects in vivo [113]. MCL blocked the growth of B16F10 melanoma cells in mice.

### 2.15. Methionine-Enkephalin

The level of the pentapeptide methionine-enkephalin was reduced in melanocytic tumors compared to non-tumor cells [3,114]. Methionine-enkephalin reduced B16 melanoma cell growth in mice, and this effect occurred by regulating the immune response and inducing a cytotoxic effect in melanoma cells [3,115]. In fact, this peptapeptide exerted an anti-melanoma action against B16 melanoma cells in vitro and in vivo because methionine-enkephalin reduced the number of melanoma cells in G2/M and S phases, promoted cell cycle arrest (G0/G1 phases), and increased the expression of opioid receptors in B16 melanoma cells [116]. Similar results were observed in A375 melanoma cells after treatment with methionine-enkephalin [117]. Moreover, in in vivo experiments, the pentapeptide decreased weight and tumor volume; increased survival; CD4^+^ to CD8^+^ T cell ratio was increased in mice treated with methionine-enkephalin; and plasma levels of interferon-γ, tumor necrosis factor-α, and interleukin-2 increased [116]. The immune response modifier imiquimod upregulated the opioid growth factor receptor facilitating the anticancer action mediated by methionine-enkephalin [3,118]. This modifier has been used to treat melanomas with good results [3,119,120,121] and its topical administration (for melanoma cutaneous metastasis) also showed good results; it was well tolerated and safe [3,122].

### 2.16. Neuropeptide Y

Neuropeptide Y expression has been reported in human samples of primary cutaneous melanoma and melanocytic nevi, but not in melanoma metastases [85]. In cutaneous melanoma this expression was higher than in melanocytic nevi and an association between the expression of neuropeptide Y and the presence of metastasis was observed [85]. Another study performed in human primary cutaneous melanomas demonstrated that thinner tumors were related to a higher expression of neuropeptide Y and that tumors showing a low neuropeptide Y expression were associated with an intense proliferation of cells, low E-cadherin expression, and high density of peritumoral mast cell infiltrates [123]. Accordingly, a high expression of neuropeptide Y is related with a better prognosis and outcome.

Obese C57BL/6 mice (fed with a high-fat diet) and control animals were injected with B16F10 melanoma cells [124]; obese mice showed a higher tumor weight than that reported in control mice, and treatment with the neuropeptide Y2 receptor antagonist BIIE0246 reduced tumor weight in obese animals, but no effect was reported in the control group. Moreover, in obese mice treated with the antagonist a decrease in serum vascular endothelial growth factor level and angiogenesis was observed, but serum nitric oxide and vascular endothelial growth factor receptor 1 levels did not change. In sum, neuropeptide Y2 receptor antagonists, by targeting angiogenesis processes, blocked melanoma growth in obese mice [124]. Chemical sympathectomy (using the neurotoxin 6-hydroxydopamine hydrobromide) decreased melanoma tumor weight in C57BL/6J mice injected with B16F10 melanoma cells [125]. Moreover, in tumors from sympathectomized animals an increased gene expression was observed regarding factors related to apoptosis (caspase 3, bcl-2), hypoxia (hypoxia-inducible factor), adrenergic and glucocorticoid receptors, tyrosine hydroxylase and neuropeptide Y. This means that sympathectomy changes the microenvironment of the tumor, decreasing melanoma growth, and this is important for the response of patients with cancer to interventions involving sympathetic signaling in both tumor and microenvironment.

### 2.17. Neurotensin

SR-48,692 (neurotensin 1 receptor antagonist) promoted cell cycle arrest and apoptosis in melanoma cells (A375) expressing neurotensin 1 receptors [126]. This expression was high in melanoma cells but low in HaCaT cells (a normal immortalized human keratinocyte cell line) [126]. Thus, SR-48,692 decreased melanoma cell proliferation and, in addition, inhibited tumor growth in mouse models [126].

### 2.18. Oxytocin

Oxytocin, through the β-arrestin 2-mediated ERK-vascular endothelial growth factor-matrix metalloproteinase 2 signaling pathway, promoted the lung metastasis of melanoma cells [127]. An upregulation of oxytocin receptors was reported in malignant melanomas; their activation favored angiogenesis and melanoma cell migration and invasion, but not melanoma cell proliferation in in vivo and in vitro experiments, through the above-mentioned signaling pathway [127]. Chronic restraint stress increased oxytocin plasma levels, favored lung metastasis of melanoma cells, and decreased survival in C57BL/6 mice; these effects were counteracted by knocking down β-arrestin 2 or the oxytocin receptor [127]. Thus, oxytocin is a prometastatic melanoma agent.

### 2.19. Somatostatin

Somatostatin 1 (96%), 2 (83%), 3 (61%), 4 (57%) and 5 (9%) receptors have been detected in human malignant melanoma samples [128]. Metastatic and primary human cutaneous melanoma cell lines expressed somatostatin 1, 2, 3 and 5 receptors and somatostatin analogs (SOM230, octreotide) did not significantly block melanoma growth or promoted the death of these cells [129]. Somatostatin 2 and 5 receptors have been observed in human uveal melanomas and the five somatostatin receptors in uveal melanoma cell lines (OCM-1, OCM-3) [130]. Somatostatin 2 receptor mRNA was higher expressed than somatostatin 5 receptor mRNA in uveal melanoma tissues, but in uveal melanoma cell lines somatostatin 2 receptors showed a lower expression than that observed for somatostatin 5 receptors, although both receptors were strongly expressed [130]. Other studies reported somatostatin 2, 3 and 5 receptors in human samples of uveal melanoma; all uveal melanomas expressed somatostatin 2 receptors; a high somatostatin 2 receptor expression was related to a better ad vitam prognosis, and somatostatin analogs (vapreotide, octreotide) inhibited the proliferation of uveal melanoma cells (Mel270, OCM3, OMM2.3) [131]. Using autoradiography, another study demonstrated a strong expression of somatostatin 2 receptors in uveal melanoma metastatic cell lines (OMM1, OMM2.3), but this was not found in cell lines derived from primary uveal melanomas (Mel92.1 and OCM-1; except in Mel202 in which a very low expression of somatostatin 2 receptors was observed); however, all metastatic and primary uveal melanoma cell lines expressed mRNA for the somatostatin 2 receptor using quantitative real-time RT-PCR [132]. In this study, only 3/14 primary samples of uveal melanoma expressed somatostatin 2 receptors and this expression was not related to tumor-free survival or any prognostic factor studied [132].

Somatostatin receptor expression in melanoma cells allowed the use of a paclitaxel formulation of solid lipid nanoparticles modified with Tyr-3-octreotide (PSM, octreotide is a somatostatin receptor agonist) to fight melanoma [133]. PSM promoted apoptosis and reduced invasion in B16F10 melanoma cells, decreased tumor volume, favored a systemic immune response, and decreased the number of nodule formations in a lung metastasis experimental animal model [133]. Thus, PSM exerted anti-melanoma effects without toxicity. In the same way, Ga-68-DOTATOC PET/CT is an essential tool to reveal meningeal metastasized uveal melanomas; hence PET imaging with 68-labeled somatostatin receptor analogs is a useful tool for staging these melanomas [134].

### 2.20. Substance P

This peptide, the natural ligand of the neurokinin-1 receptor, is expressed in metastatic melanomas, primary invasive malignant melanomas, melanomas in situ, spindle and epithelioid cell (Spitz) nevi, and atypical nevi, but it was not found in benign melanocytic nevi [135]. Substance P modulates tumor growth via immune mechanisms in a mouse experimental model (animals were injected with K1735 melanoma cells) [136]. Implanted pumps delivered a continuous administration of substance P in mice and later, five days after implantation, animals received K1735 cells. Pretreatment with substance P delayed the growth of tumors, but this effect did not occur when mice were depleted of T or natural killer cells and, importantly, the substance P protection property can be transferred from cells treated with substance P when they were administered to mice that did not receive substance P pretreatment [136]. This study showed that pretreatment with substance P prevented/delayed tumor development in vivo, that the involvement of both T and natural killer cells was needed in this process, and that treatment with substance P before tumor development favored the action of immune mediators which exerted a protective effect against melanoma.

Substance P, after binding to neurokinin-1 receptors, blocked melanogenesis in mouse B16F10 melanoma cells [137]. When C5BL/6 mice containing previous melanoma cells were treated with ionizing radiation (45Gy), tumor growth was blocked, survival was increased and the level of substance P in the tumor and surrounding skin decreased [138]. Radiotherapy also promoted systemic changes in substance P levels, reducing these levels in the skin located far from the site of radiation application, as well as in the adrenal gland and lung [138]. Melanoma cell growth was blocked when mouse B16F10 and B16LNAD (made from metastatic lesions) melanoma cells were treated with substance P, and, in addition, this effect potentiated the inhibitory action mediated by radiotherapy [138]. Thus, it seems that the decrease observed in the level of substance P due to radiotherapy may underlie melanoma radioresistance [138]. Another study has reported that substance P promoted apoptosis in B16F10 melanoma cells in vitro [139]. These findings are contradictory, as other studies have reported the opposing effects of substance P. For example, L-732,138 (neurokinin-1 receptor antagonist) promoted apoptosis in human melanoma cells (COLO 679, COLO 858, MEL HO) and inhibited the substance P mitogen stimulation of melanoma cells because this study also demonstrated that substance P promoted the proliferation of melanoma cells [140]. The antitumor effect of the neurokinin-1 receptor antagonist L-733,060 has also been demonstrated in human melanoma cell lines (COLO 679, COLO 858, MEL HO); this antagonist inhibited, in a dose-dependent manner, the growth of the three cell lines [2]. Another study showed that melanoma cells (COLO 679, COLO 858, MEL HO) expressed the neurokinin-1 receptor (this was observed in all human samples and melanoma cell lines studied); that melanoma cell lines expressed mRNA for this receptor; that the neurokinin-1 receptor was involved in the viability of melanoma cells; that the neurokinin-1 receptor antagonist aprepitant, in a concentration-dependent manner, inhibited melanoma cell growth; and that melanoma cell death was due to apoptotic mechanisms [141]. Moreover, another study demonstrated that the neurokinin-1 receptor was overexpressed in COLO 679, COLO 858, and MEL HO melanoma cell lines as well as the TACR1 gene which codes the neurokinin-1 receptor [142]. In the previous study, neurokinin-1 receptors were observed in all human uveal melanoma samples studied and, in addition, the immunosuppressive agent cyclosporin A blocked (mediated by the neurokinin-1 receptor) COLO 679, COLO 858, and MEL HO cell growth and promoted apoptosis in these cells [142]. Previous findings suggest that the substance P/neurokinin-1 receptor system plays a crucial role in melanoma development as for this reason the neurokinin-1 receptor has been proposed as a melanoma target [143]. Accordingly, the dual (oncogenic and anti-melanoma) effect of substance P in melanoma should be studied in greater depth and the molecular mechanisms involved should be clearly determined.

### 2.21. Thyrotropin-Releasing Hormone

Thyrotropin-releasing hormone (TRH) bound to human melanocortin 1 receptors expressed in mouse B16 melanoma cells, but did not bind to human melanocortin 3, 4 and 5 receptors [144]. Moreover, TRH analogs (e.g., TRH-Gly-NH2, TRH-OH) did not bind to any of the melanocortin receptors tested [144]. TRH and α-melanocyte-stimulating hormone promoted the synthesis of cAMP in B16 melanoma cells reaching the same maximum levels, and TRH was docked into a binding pocket of a melanocortin 1 receptor molecular model at only a little higher energy than a short cyclic melanocyte-stimulating hormone peptide [144]. This study demonstrated that both TRH and melanocyte-stimulating hormone activated in a similar way the melanocortin 1 receptor. TRH (mRNA and protein) have been detected in melanoma cell lines (A375, TXM18, MeWo, WM35, WM793); TRH immunoreactivity was observed in benign nevi (51.9%), dysplastic nevi (69.7%) and melanomas (63%); and TRH expression was higher in dysplastic nevi than in benign nevi, and this could serve as a predictive tool for melanoma development [145]. Moreover, TRH favored the proliferation of melanoma cells, but this was not observed in melanocytes [145].

### 2.22. Urocortin

Ultraviolet-B-radiation-stimulated urocortin-1 favored the production of tyrosinase-related protein 1 through Nur77/Nurr-1 transcription factors in human melanoma HMV-II cells [146]. Thus, ultraviolet B radiation augmented the protein level of tyrosinase-related protein 1, increased Nur77/Nurr-1 and urocortin 1 mRNA levels, and urocortin 1 knockdown blocked the ultraviolet-B-radiation-induced increase in the protein level of tyrosinase-related protein 1 [146]. Thus, it seems that urocortin 1 synthesized in melanoma cells exerted an autocrine action.

### 2.23. Vasoactive Intestinal Peptide

The effect of the vasoactive intestinal receptor antagonist ANT308 (VPAC1 and VPAC2 receptor antagonist) has been tested in melanoma cells (Mel 290, B16LS9, HT-144, B16F10) in in vitro and in vivo experiments [147]. This antagonist inhibited melanoma cell proliferation and migration, promoted apoptosis, decreased N-cadherin/melanoma cell adhesion molecule expressions, and reduced tumor volume and the number/size of liver metastases [147]. VPAC2 receptor knockdown showed the same actions regarding cell proliferation and migration as those observed when ANT308 was administered [147]. Thus, this study demonstrated that the vasoactive intestinal peptide/VPAC receptor system is involved in melanoma progression and liver metastasis.

### 2.24. Vasopressin

A study has reported the lack of vasopressin in malignant melanomas [148], and desmopressin, a synthetic vasopressin analog, decreased melanoma lung metastasis (B16 cells) in transgenic mice overexpressing the tissue inhibitor of metalloproteinases 1 [149]. This decrease was not due to cytotoxic effects on melanoma cells, and no vasopressin receptor was detected in B16 melanoma cells [149]. Thus, it seems that high levels of circulating tissue inhibitor of metalloproteinases 1 exerted a co-operative role in the anti-melanoma effect exerted by desmopressin.

### 2.25. Other Peptides

Recently, anti-melanoma strategies have been developed using other peptides. Thus, the synthetic alpha-helical peptide KW18 showed minimal toxicity against fibroblasts but a potent toxicity against resistant melanoma cells. KW18 regulated cell cycle and promoted apoptosis in these melanoma cells; hence it is a safe, selective and stable therapeutic tool for the treatment of drug-resistant melanomas [150]. Cationic peptides, targeting the plasma membrane of tumor cells, exerted cytotoxic effect against melanoma cells (SK-MEL-28) [151] and the peptide PEPAD decreased cell migration and promoted apoptosis in B16F10-Nex2 melanoma cells [152]. The immunomodulating peptide IK14004 inhibited lung melanoma progression in a mouse experimental model (C57BL/6) without compromising immune tolerance [14]. The proteolysis-resistant d-dodecapeptide ^D^PMI-ω disrupted the p53-MDM2-MDMX complex by antagonizing MDMX and MDM2 oncogenic proteins and blocked B16 melanoma cell growth in vivo and, when ^D^PMI-ω and anti-PD-1 antibodies were co-administered, the efficacy of the immunotherapy increased by inhibiting CD4^+^/CD25^+^ regulatory T cells and expanding CD3^+^/CD8^+^ cytotoxic T cells [153]. Caerin peptides (1.1 (F1) and 1.9 (F3) from Australian tree frogs) blocked B16 melanoma cell proliferation in vitro by activating immune mechanisms (e.g., macrophage infiltration and pro-inflammatory cytokine expression were augmented, increasing the anti-melanoma response) and downregulating lipid metabolites (e.g., unsaturated fatty acid synthesis and fatty acid biosynthesis were blocked) [154]. Moreover, caerin peptides increased the level of 3-hydroxyvalproic acid and carnitine derivatives involved in anti-inflammatory and antiproliferative actions [154].

Other peptides are used in targeted drug delivery systems to treat melanoma [155]. In this sense, the pYEEIE peptide-functionalized, rhodiola-rosea-derived exosome-like nanovesicles loaded with the chemotherapeutic drug doxorubicin (pYEEIE-RELNs-DOX) has been developed. The administration of pYEEIE-RELNs-DOX in mice with melanoma showed a higher antitumor effect by blocking melanoma growth when compared with the administration of free doxorubicin, and, in addition, no toxicity was observed in the kidneys, lungs, spleen, liver and heart; however, free doxorubicin promoted heart tissue injury [155]. Thus, pYEEIE-RELNs-DOX is a low-toxicity targeted delivery system to fight melanoma. Moreover, the administration of hydrolyzed polyacrylonitrile nanofibers biofunctionalized with the dipeptide L-carnosine and loaded with bio CAR-synthesized zinc oxide nanoparticles (ZCPAN) for postsurgical melanoma treatment has been reported [110]. ZCPAN promoted oxidative injury and apoptosis in melanoma cells (A375-M2); hence it seems that this strategy is quite promising to block melanoma regrowth after skin tumor surgical procedures.

Table 1 and Table 2 respectively show a summary of the oncogenic (Table 1) and anti-melanoma (Table 2) peptides regulating melanoma progression.

## 3. Discussion

Some peptidergic systems exert an oncogenic effect (adrenomedullin, hemokinin-1, oxytocin, and thyrotropin-releasing hormone), others an anti-melanoma action (calcitonin gene-related peptide, β-endorphin, gonadotropin-releasing hormone, kisspeptin, melittin, methionine-enkephalin, neuropeptide Y, and somatostatin), and others a dual oncogenic and anti-melanoma effect (angiotensin, bradykinin, corticotropin-releasing hormone, endothelin, α-melanocyte-stimulating hormone, and substance P) in melanoma (Table 1 and Table 2). Caerin and cationic peptides and others such as KW18, IK14004 and ^D^PMI-ω also exert anti-melanoma actions (Table 2). Previous data demonstrate the enormous number of peptides and the functional complexity of the peptidergic systems involved in melanoma development, but at the same time the data show the large and varied therapeutic potential currently available to combat melanoma. Therefore, peptidergic systems open the field to numerous lines of research which, if continued and properly planned, will lead to promising results that could be translated into clinical practice. In fact, the research carried out to date shows many anti-melanoma strategies ranging from peptide receptor antagonists to vaccines, including viruses and bacteria (Table 3). In this section, peptidergic systems and anti-melanoma treatments, oncogenic/anticancer peptides in melanoma, peptide receptors, peptidergic systems, melanoma risk and immune system relationships, peptidergic systems and delivery strategies, peptidergic systems and research lines in melanoma, peptide receptor agonists and antagonists with anti-melanoma properties in melanoma will be mentioned and discussed.

### 3.1. Peptidergic Systems and Anti-Melanoma Treatments

Table 3 shows the numerous peptides serving as potential anti-melanoma therapeutic targets and demonstrates the great potential and diversity of the antitumor strategies that currently exist to combat melanoma, for example, anti-adrenomedullin antibodies [16], anti-adrenomedullin receptor antibodies [16], mRNA vaccines [17], angiotensin I-converting enzyme inhibitors [23], angiotensin II type 2 receptor antagonists [24], anti-E-selectin antibodies [23], kinin B1 receptor agonists [30], endothelin B receptor antagonists (A-192,621) [44], MAPK blockade [47], rendomab B4 antibodies [48], endothelin-3 silencing [54], gonadotropin-releasing hormone receptor agonists [61], neurokinin-1 receptor antagonist [63], beta (1)-integrin subunit antibodies [74], melanocortin 1 receptor agonists-camptothecin [13], melanocortin 4 receptor antagonists-vemurafenib [91], diallyl trisulfide [107], hepatitis B core virus-like particles [110], melittin-RADA32-CpG-lysate vaccine [113], neuropeptide Y2 receptor antagonists [124], neurotensin 1 receptor antagonists [126], and vasoactive intestinal receptor antagonists [147]. It is true that some of these strategies need to be investigated further, but they show the plethora of anti-melanoma strategies that currently exist and that should be further strengthened and developed. In sum, Table 3 shows seventeen peptidergic systems targeted with different strategies favoring anti-melanoma effects. Table 3 also illustrates the complexity of possible treatments for melanoma due to the large number of peptidergic systems involved in melanoma progression. One of the most common treatments is the use of peptide receptor antagonists: angiotensin II type 2 receptor antagonists (EMA401/PD-123,319), endothelin B receptor antagonists (BQ788, A-192,621), neurokinin-1 receptor antagonists (aprepitant, L-732,138, L-733,060), melanocortin 4 receptor antagonists (ML00253764), neuropeptide Y2 receptor antagonists (BIIE0246), neurotensin 1 receptor antagonists (SR-48,692), and vasoactive intestinal receptor antagonists (ANT308) [2,24,44,56,63,91,124,126,140,147]. This is a crucial line of research that needs to continue in the future as well as the study of peptide receptor agonists with anti-melanoma properties: kinin B1 receptor agonists (DABK), gonadotropin-releasing hormone receptor agonists (zoralex), and melanocortin 1 receptor agonists (melanotan-II) [13,30,32,61,62]. Anti-melanoma strategies (e.g., peptide receptor antagonists and anti-melanoma peptides) could be applied in combination with standard treatments used in clinical practice (surgery, chemotherapy, radiotherapy, and immunotherapy). For example, since peptide receptor antagonists/anti-melanoma peptides block the migration of melanoma cells and promote apoptosis in these cells, a possible combination therapy would be the administration of these antagonists before and after surgical procedures to reduce tumor volume and prevent migration. In fact, current data confirm other combination therapies with excellent results: kisspeptin 54 increased vemurafenib (B-rafV600E inhibitor) pro-apoptotic action in vemurafenib-resistant melanoma cells [67]; the melanocortin 4 receptor antagonist ML00253764 in combination with vemurafenib favored an antitumor synergic action against melanoma cells [91]; and co-administration of endothelin B receptor antagonists and MAPK inhibitors is a promising treatment for patients with melanoma and MAPK signaling pathway hyperactivation because this strategy decreased tumor growth and increased survival [46]. This is important because after treatment with MAPK inhibitors, most tumors developed resistance in melanoma patients with BRAF mutations and because the endothelin/endothelin B receptor system did not promote resistance towards MAPK inhibitors. Combination therapies need to be rigorously studied, strengthened and explored in greater depth since many of them have not been studied, including those shown in Table 3.

Unlike what happens with the peptidergic systems in other types of cancer, a large number of anti-melanoma strategies have been published to fight this disease. This is the case of vaccines, antibodies, CAR-macrophages and microRNAs (Table 3). Accordingly, vaccines killed melanoma cells, decreased tumor volume and weight, reduced the number of blood vessels and the size and number of lung metastases, increased the number of CD8^+^ T cells without inducing an immunosuppressive tumor microenvironment, and activated dendritic cells favoring the infiltration of cytotoxic T lymphocytes in the tumor microenvironment, and the combination of vaccines showed a more effective action than vaccines given separately [17,18,60,113]. Antibodies (rendomab-B4, xiRB49-NMAE) blocked melanoma cell migration and exerted antitumor actions against melanoma cells and tumors [48,50], whereas CAR-macrophages exerted high anticancer activity against melanoma cells expressing a high number of endothelin B receptors, but, importantly, no activity was observed against melanoma cells expressing a low number of these receptors [51]. This is a crucial finding which implies a very specific antitumor action against melanoma cells expressing a large number of these receptors. The involvement of microRNAs in melanoma progression has also been studied; microRNA Let-7i promoted apoptosis in tumor cells, counteracted melanoma cell proliferation and migration, and decreased the expression of proliferation/metastasis-related genes, and it was downregulated in patients with melanoma [65].

### 3.2. Oncogenic and Anticancer Peptides in Melanoma

Melanoma cells expressed a higher number of neurotensin 1 receptors than normal immortalized human keratinocyte cells [126]. This is crucial for the application of specific and safe anti-melanoma strategies because peptides through their respective receptors expressed/overexpressed in melanoma cells; favored cell proliferation, migration, invasion and metastasis; and promoted lymphangiogenesis and angiogenesis [3,16,22,23,24,39,43,63,127]. These peptides act as autocrine, paracrine and endocrine agents, which means that anti-oncogenic peptide antibodies or anti-melanoma peptide receptor antagonists could block all the oncogenic effects mentioned above, as it happens [16,23,24,63]. For example, ANT308 (vasoactive intestinal receptor antagonist) blocked melanoma cell proliferation and migration, promoted apoptosis, decreased N-cadherin/melanoma cell adhesion molecule expressions and reduced tumor volume and the number/size of liver metastases [147]. That is, the same antagonist favored a plethora of anti-melanoma actions. An interesting finding should be explored further and the molecular mechanisms involved should be well understood: TRH favored the proliferation of melanoma cells, but this was not observed in melanocytes [156]. This would help better understand how peptidergic systems regulate melanoma progression.

Peptides such as calcitonin gene-related peptide, β-endorphin, gonadotropin-releasing hormone, endothelin-3, melittin, α-melanocyte-stimulating hormone and CRH also exerted anti-melanoma effects (e.g., apoptosis, cell proliferation decrease, migration, invasion and metastasis inhibition, tumor weight/volume decrease, anti-angiogenic effect, survival increase, caspase 3/9 activity increase, and PI3K/Akt/mTOR and MAPK pathways downregulation) [3,35,36,54,61,62,73,106,108,115,116]. Peptide analogs can also exert anti-melanoma actions; this is the case of the synthetic vasopressin analog desmopressin which reduced melanoma lung metastasis [149]. It is important to remark that, compared with control animals, melanoma developed in mice increased the synthesis of hypothalamic kisspeptin which exerts anticancer (antiproliferative) and antimetastatic effects [64], and, in a similar way, a positive association between tumor progression and β-endorphin expression (decreases tumor growth and increases immune cell infiltration) has been reported in human melanoma tissues [41]. Both examples could represent an important endogenous mechanism in response to tumor development by favoring the synthesis of anti-melanoma peptides to fight melanomas, and it is important to delve deeper into the molecular mechanisms involved. In addition, one of the previous studies showed that daylight exposure favored a higher hypothalamic kisspeptin expression and tumor growth speed [64]. In this sense, it is also known that ultraviolet B radiation favored the synthesis and release of α-melanocyte-stimulating hormone from melanoma cells [72] and that the α-melanocyte-stimulating hormone/melanocortin 1 receptor system decreased the risk of melanoma development by preserving melanocytes’ genomic stability, via controlling the DNA damage response to solar ultraviolet radiation [71]. This is important because DNA repair ability is essential to regulating melanoma risk. However, it should be noted that α-melanocyte-stimulating hormone also exerts a dual action on melanoma cells; it promotes the proliferation of these cells [104] and blocks the migration of melanoma cells [59,74], hence the importance of understanding the molecular mechanisms that control these processes in order to better understand the specific action of α-melanocyte-stimulating hormone or other peptides in melanoma and to determine whether the release of α-melanocyte-stimulating hormone mediated by ultraviolet B radiation is favorable or unfavorable to melanoma progression. This dual oncogenic and anti-melanoma effect also occurs with other peptides. For example, the effects of bradykinin on melanoma cells are contradictory: kinin B1 receptor activation counteracted the growth/metastasis of melanoma but bradykinin favored the migration and invasion of melanoma cells via the synthesis of nitric oxide and superoxide [29,30,33]. Substance P prevented/delayed melanoma development [136] and favored apoptosis in melanoma cells [139], but other studies showed that neurokinin-1 receptor antagonists inhibited the substance P mitogen stimulation of melanoma cells [140]. The fact that the same peptide exerts a dual oncogenic or anti-melanoma action may be due to multiple factors: different melanoma cell lines studied belonging to the same species (e.g., murine or human) or to different species, experimental models used, receptor types expressed by melanoma cells, and the G protein types and signaling pathways involved in the processes. For example, depending on the angiotensin II type receptor involved, angiotensin II exerted an oncogenic or an anticancer action in melanoma [24]. In general, contradictory findings could also be due to several other reasons: variability based on tumor stage or microenvironment, reliance on various testing models and/or experimental conditions.

Research should be developed in melanoma to know how oncogenic peptides interact with each other, how anti-melanoma peptides interact with each other, how anti-melanoma peptide receptor antagonists interact with each other, how oncogenic and anti-melanoma peptides interact with each other, how anti-melanoma peptide receptor antagonists interact with anti-melanoma peptides, and how oncogenic peptides and anti-melanoma peptide receptor antagonists interact. An understanding of these interactions will be of great help in knowing how various peptidergic systems regulate melanoma development (e.g., by increasing/decreasing oncogenic/anti-melanoma effects and by favoring synergistic effects) and in developing more specific anti-melanoma strategies. This is crucial since, for example, an immunostaining positive correlation between galanin and α-melanocyte-stimulating hormone has been reported in melanomas, but the functional meaning is currently unknown [80]. As indicated above, α-melanocyte-stimulating hormone exerts a dual action on melanoma, and the effect exerted by galanin on the tumor is currently unknown. This also indicates that the oncogenic/anticancer actions of numerous peptidergic systems in relation to melanoma is unknown (e.g., oncogenic and/or anti-melanoma effect, peptide receptor expression, and peptide receptor viability); therefore a great deal of basic research is needed. And there is another important finding: methionine-enkephalin increased the expression of opioid receptors in melanoma cells [116]; hence the use of imiquimod which upregulated the expression of peptide receptors could significantly improve the anti-melanoma effects mediated by methionine-enkephalin [3,118,119,120,121,122]. This is an important finding that must be exploited and shows how peptidergic systems regulate the expression of peptide receptors.

In addition to that mentioned above, there are other peptides that have recently demonstrated their actions against melanoma. For example, KW18 showed minimal toxicity against fibroblasts but a potent toxicity against drug-resistant melanoma cells (apoptosis) [150]; cationic peptides exerted cytotoxic effects against melanoma cells [151]; PEPAD decreased cell migration and promoted apoptosis in melanoma cells [152]; IK14004 inhibited lung melanoma progression without compromising immune tolerance [14]; and ^D^PMI-ω blocked melanoma cell growth [153]. These are some examples of the new peptides that are being studied as anti-melanoma agents, greatly expanding the range of compounds with anti-melanoma action.

### 3.3. Peptide Receptors in Melanoma

The overexpression of peptide receptors can be used for diagnostic and therapeutic purposes in melanoma. For example, melanocortin 1 receptor is overexpressed in melanoma cells, and it can be targeted by ligand drug conjugates by coupling the melanocortin 1 receptor agonist to cytotoxic drugs (e.g., melanotan-II-camptothecin reduced melanoma cell growth) [13], by the preparation of ^67^Ga-labeled NODAGA-conjugated lactam-cyclized α-melanocyte-stimulating hormone as a radiometal chelator for α-melanocyte-stimulating hormone radiolabeling [94] or by using peptide receptor antagonists or anti-melanoma peptides. This overexpression, compared to normal cells, is a very useful tool because it could increase the specificity and safety of the anti-melanoma treatments.

To understand the interactions between the peptides mentioned above, it is important to know that different peptides can bind to the same receptor; this is the case of TRH and α-melanocyte-stimulating hormone which activated the melanocortin 1 receptor in a similar way [144]. This is another essential example of the field that remains to be studied and that will help to understand the interactions between peptidergic systems in melanoma. Moreover, peptide receptor polymorphisms are also crucial for the response of melanoma cells to peptides since the invasion of melanoma cells transfected with the wild-type melanocortin 1 receptor was blocked with α-melanocyte-stimulating hormone, but melanoma cells did not respond to α-melanocyte-stimulating hormone when these cells displayed melanocortin 1 receptor polymorphisms [75]. Moreover, CAR-macrophages exerted a high anticancer activity against melanoma cells expressing a high number of endothelin B receptors, but no activity was observed against melanoma cells expressing a low number of these receptors [51]. This is an important finding regarding the expression of peptide receptors. Finally, another important finding that must be investigated in-depth: the antibody rendomab B1 showed antagonist properties against endothelin B receptors expressed in endothelial cells but had a poor affinity for these receptors expressed in melanoma cells [48]. This observation is important because it suggests a tumor-specific endothelin B receptor form.

### 3.4. Peptidergic Systems and Melanoma Risk

The expression of the peptidergic systems is useful for diagnosis and prognosis. For example, melanocortin 1 receptor expression could be a marker for melanoma progression (this expression has been associated with shorter survival in metastatic and primary melanomas) [87] and a predictive factor for postoperative outcomes in patients suffering from melanomas, because patients with melanoma showing a low melanocortin 1 receptor expression had a better prognosis than those with high expression [89]. Moreover, melanocortin 1 receptor variants have been related with an increased cutaneous melanoma risk [92]. Other examples: an association between neuropeptide Y expression and the presence of metastasis has been reported, thinner primary cutaneous melanomas were related with a higher expression of neuropeptide Y, and tumors showing a low neuropeptide Y expression were associated with an intense proliferation of cells and low E-cadherin expression [85,123]. Thus, high neuropeptide Y expression has been associated with a better prognostic and outcome, and the data suggest that this peptide exerts anti-melanoma effects. This must be confirmed. Finally, all lymph node biopsies of the melanoma patients studied were immunohistochemically positive to rendomab 49 antibody, suggesting that this antibody could be a useful tool for the diagnostic of these patients [50].

### 3.5. Peptidergic and Immune Systems

Another crucial role played by the peptidergic systems in melanoma is their relationships with the immune system and tumor microenvironment, since peptides regulate both the tumor microenvironment and immune system. In this sense, the therapeutic efficacy of blocked anti-programmed death-1 antibodies was re-established by inhibiting the angiotensin system [24]; this is important because by increasing the anticancer actions of anti-programmed death-1 antibodies, tumor-induced immunosuppression is reversed. In the melanoma microenvironment, it has been reported that endothelin-3 overexpression exerted (mediated by endothelin B receptors) an immunosuppressive effect favoring the escape of melanoma cells from tumor immunity [45]. α-Melanocyte-stimulating hormone decreased the interactions between melanoma cells and T lymphocytes, also promoting the escape of tumor cells from the immune system [80], and melanocortin 1 receptor activation in melanoma cells impaired tumor T cell infiltration in the tumor microenvironment, reducing anticancer immunity; hence T cell infiltration blockade counteracted antitumor immunity and favored resistance to immune checkpoint blockade therapies [90]. The loss of melanocortin 1 receptors in melanoma cells favored T cell anticancer response, and the resistance to the immune checkpoint blockade was overcome [90].

The synthetic peptide melittin–dKLA favored apoptosis in M2-like tumor-associated macrophages, and the blockade of M2 macrophage proliferation and migration by this peptide was associated with a decrease in melanoma tumor growth [109]. Mertansine conjugated with melittin promoted apoptosis in M2 tumor-associated macrophages and decreased M2-like tumor-associated macrophages leading to the blockade of melanoma cell growth, migration and invasion, improving the survival rate and increasing natural killer/CD8^+^ cytotoxic T cell infiltration in the tumor microenvironment [1]. When ^D^PMI-ω and anti-PD-1 antibodies were co-administered, the efficacy of the immunotherapy increased by inhibiting CD4^+^/CD25^+^ regulatory T cells and expanding CD3^+^/CD8^+^ cytotoxic T cells [153]. Finally, caerin peptides blocked the proliferation of B16 melanoma cells by increasing macrophage infiltration and the expression of pro-inflammatory cytokines [154].

### 3.6. Peptidergic Systems and Delivery Strategies

There are numerous strategies for peptide delivery in melanoma which favor tumor suppression, protect peptides from enzymatic degradation, decrease cytotoxicity, and improve tumor selectivity [110]. For example, hepatitis B core virus-like particles were developed to encapsulate the anticancer peptide melittin to treat subcutaneous melanoma and lung metastasis [110], and RGD-melittin expressed and released by Salmonella typhimurium blocked melanoma cell proliferation, migration and invasion, inhibited chemotaxis and promoted apoptosis [111]. Previous examples show the importance and high potential of microbial-mediated precision anti-melanoma strategies [110,111]. Hypochlorous-acid-treated tumor cells were loaded into a melittin-encapsulated hydrogel scaffold and administered intratumorally; this procedure augmented survival, promoted tumor cell death and cytotoxic T lymphocyte infiltration, inhibited tumor growth, and increased the anticancer effects of the immune checkpoint blockade [112]. This is one example of the importance of the delivery of anticancer agents and the generation of cell-derived secretions. The expression of somatostatin receptors in melanoma cells has allowed the use of a paclitaxel formulation of solid lipid nanoparticles modified with Tyr-3-octreotide (PSM, octreotide is a somatostatin receptor agonist) to fight melanoma [133]. PSM promoted apoptosis and reduced invasion in melanoma cells, decreased tumor volume, and favored a systemic immune response [133]. Thus, the previous strategy is based on exploiting peptide receptor expression in melanoma. pYEEIE peptide-functionalized rhodiola-rosea-derived exosome-like nanovesicles loaded with the chemotherapeutic drug doxorubicin (pYEEIE-RELNs-DOX) have been developed [155]. pYEEIE-RELNs-DOX blocked melanoma growth and no toxicity was observed in the kidneys, lungs, spleen, liver and heart; however, free doxorubicin promoted heart tissue injury [155]. Finally, the administration of hydrolyzed polyacrylonitrile nanofibers biofunctionalized with the dipeptide L-carnosine and loaded with bio (CAR)-synthesized zinc oxide nanoparticles (ZCPAN) for postsurgical melanoma treatment has been reported [110]: ZCPAN promoted oxidative injury and apoptosis in melanoma cells, and this procedure has been suggested to block melanoma regrowth after skin tumor surgical procedures.

### 3.7. Peptidergic Systems and Other Research Lines in Melanoma

According to current knowledge, there are other very interesting lines of research in melanoma regarding the peptidergic systems. Thus, peptides, as is the case with angiotensin II, can also bind to the angiotensin I-converting enzyme (overexpressed in melanoma cells) favoring the proliferation of these cells (blocked with lisinopril, ACE inhibitor) [20]. This is important because increased melanoma risk has been reported for ACE inhibitors (antihypertensive treatment) [27], and, in addition, lisinopril promoted human MV3 melanoma cell migration and invasion [21]. This must be fully investigated. The renin–angiotensin system has been associated with treatment resistance and cancer stem cells, and this means that the efficacy of therapeutic strategies (e.g., immunotherapy and targeted therapies) could be improved by regulating the paracrine renin–angiotensin system [28]. This must be investigated in-depth as well as the lower CRH expression observed in men compared to women with melanoma metastasis, and it must be confirmed whether a higher CRH expression is associated with decreased overall survival in men [36]. These findings point to sex differences in the expression of the peptidergic systems in melanoma. Chemical sympathectomy decreased melanoma tumor weight, and in tumors from sympathectomized animals an increased gene expression was observed regarding factors related to apoptosis; thus, sympathectomy changes the microenvironment of the tumor, decreasing melanoma growth, and this is important for the response of patients with cancer to interventions involving sympathetic signaling in both the tumor and microenvironment [125]. Melanoma progression, aggressiveness and angiogenesis are determined by growth factor exchanges occurring between melanoma and endothelial cells in the hypoxic tumor microenvironment; these exchanges control cell motility and vascularization via vascular endothelial growth factor and endothelin-1 by exerting autocrine/paracrine actions [44]. This is a crucial point that must be developed and also highlights the importance of the peptidergic systems in melanoma.

Another interesting research line to study is the link between obesity and melanoma [157]. The adipose tissue releases adipokines which are linked to inflammatory mechanisms, melanoma cell proliferation, migration and invasion and angiogenesis. An increase in the synthesis of lipids has been reported in melanoma cells, and these molecules are involved in oncogenic pathways affecting melanoma progression [157]. Moreover, an interesting observation which deserves further investigation has been published: obese mice showed higher melanoma tumor weights than those reported in control mice, and treatment with the neuropeptide Y2 receptor antagonist BIIE0246 reduced tumor weight in obese animals, but no effect was reported in the control group [124]. Chronic infections mediated by pathogens (*Candida tropicalis*, *Pseudomonas aeruginosa*, and *Staphylococcus aureus)* favored melanoma development by increasing the epithelial–mesenchymal transition, favoring the synthesis of pro-inflammatory cytokines and promoting chronic inflammation, leading to tumor cell invasion and metastasis [158]. This means that microbial pathogens regulate the tumor microenvironment and that antimicrobial agents could be used to control both the microenvironment and infections. Finally, it has been suggested that the decrease in the level of substance P, due to radiotherapy, may underlie melanoma radioresistance [138]; this must be studied in detail.

### 3.8. Peptide Receptor Agonists/Antagonists with Anti-Melanoma Properties

**Figure 1:**
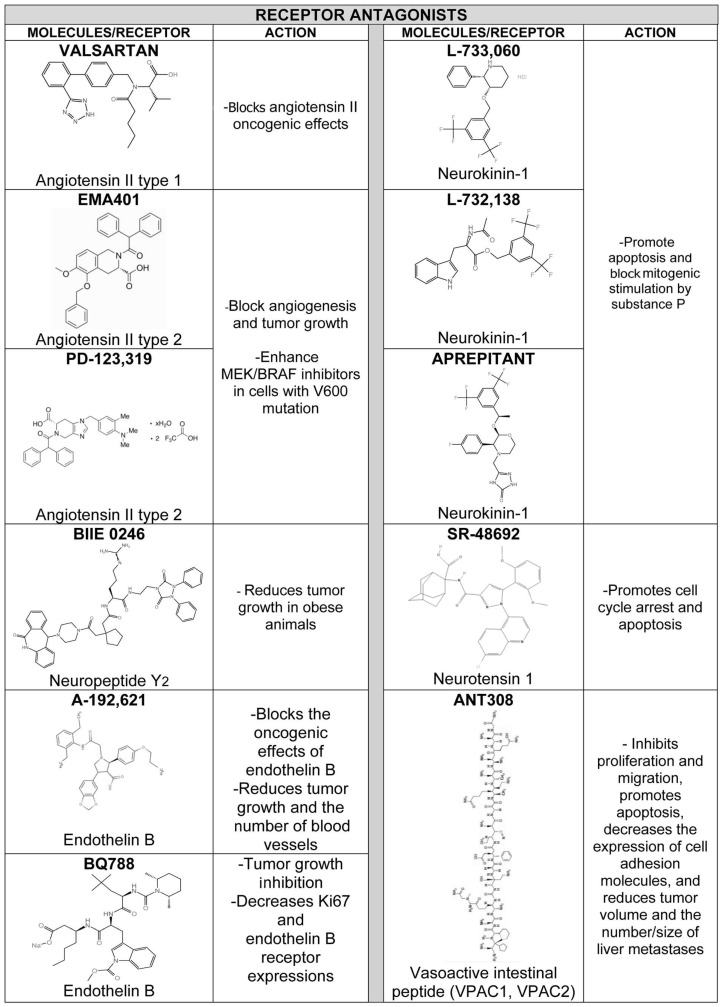

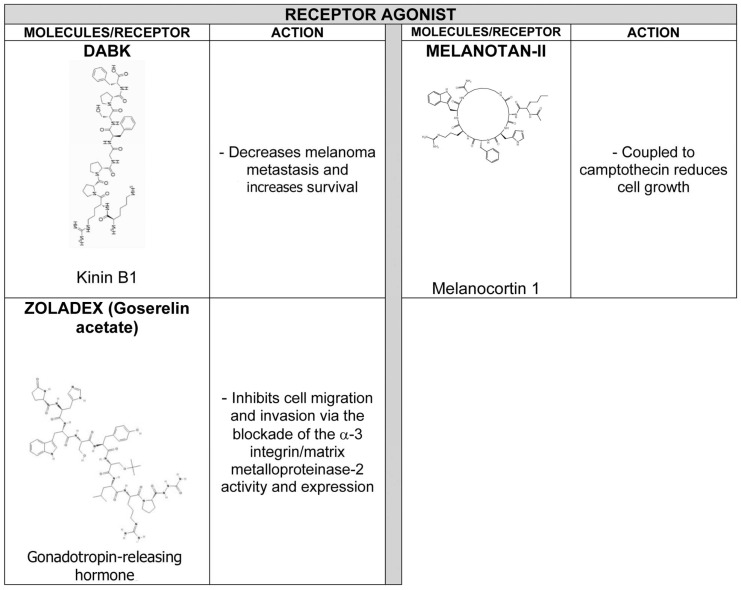
The chemical structures of peptide receptor agonists and antagonists showing anti-melanoma properties. The structures were drawn with the KingDraw software (version 3.0). The peptide receptors involved and the anti-melanoma actions exerted are also indicated [2,13,30,32,44,56,61,63,91,124,126,141,147].

Based on the above, peptide receptor agonists and antagonists have demonstrated their anti-melanoma effect in many in vitro and in vivo experiments. Valsartan (angiotensin II type 1 receptor antagonist); EMA401 and PD-123,319 (angiotensin II type 2 receptor antagonists); DABK (kinin B1 receptor agonist); zoladex (goserelin acetate, gonadotropin-releasing hormone receptor agonist); melanotan-II (melanocortin 1 receptor agonist); BIIE0246 (neuropeptide Y2 receptor antagonist); SR-48,692 (neurotensin 1 receptor antagonist); BQ788 and A-192,621 (endothelin B receptor antagonists); L-733,060, L-732,138 and aprepitant (neurokinin-1 receptor antagonists); and ANT308 (vasoactive intestinal receptor antagonist) are promising molecules for the treatment of melanoma (Figure 1) [2,13,20,23,24,30,32,44,56,61,63,91,124,126,140,147]. The effects of these molecules against melanoma need to be studied further; they should be administered in combination with other anti-melanoma therapies (e.g., chemotherapy), and the co-administration of several of these molecules (e.g., goserelin acetate and aprepitant) should also be studied in-depth.

## 4. Conclusions

**Figure 2:**
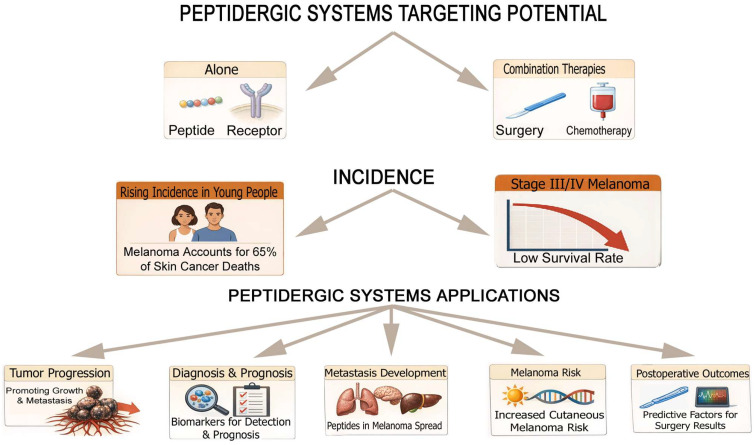
Melanoma incidence and peptidergic systems targeting potential and applications.

**Figure 3:**
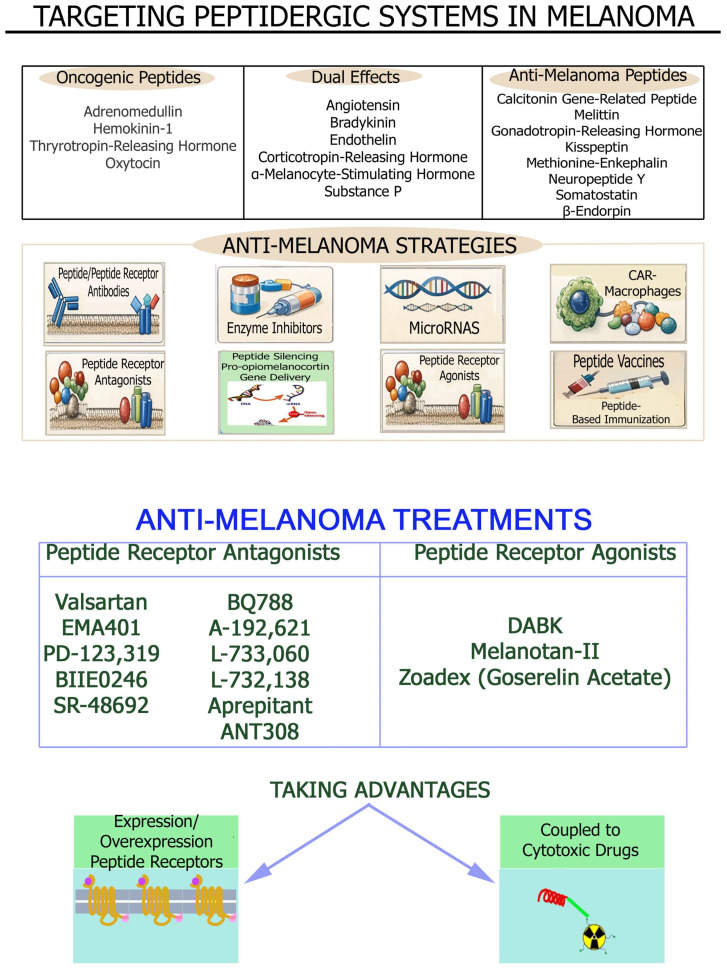
Oncogenic and/or anti-melanoma peptides and anti-melanoma strategies.

**Figure 4:**
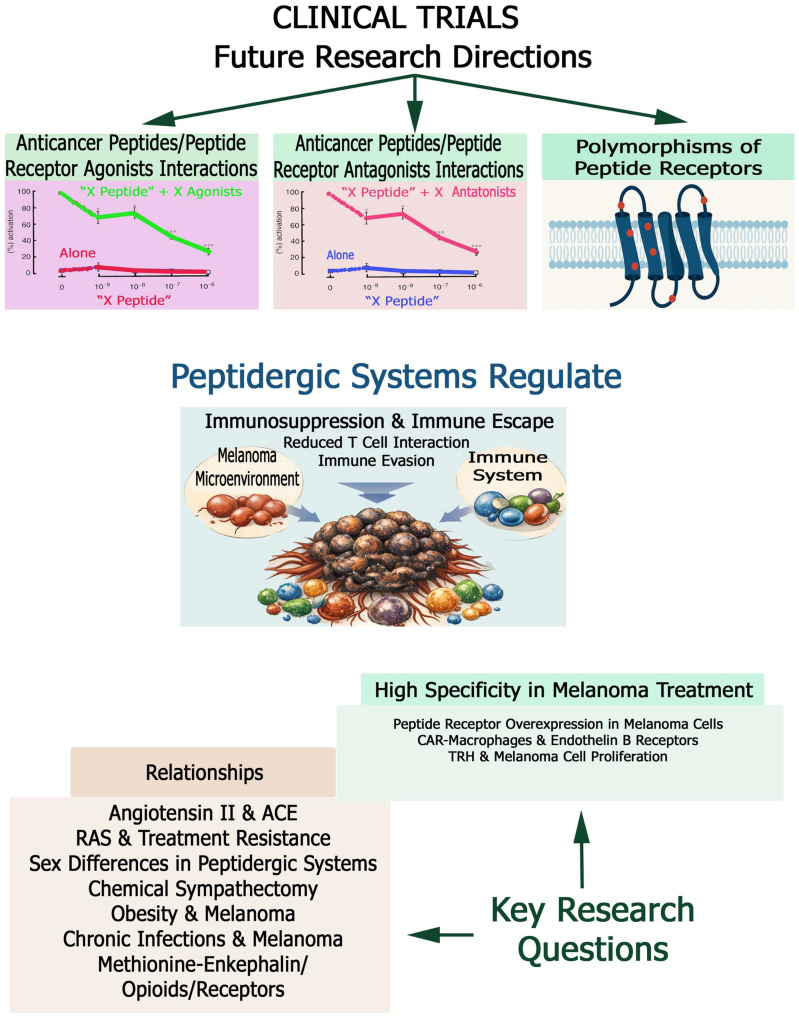
Future research directions and key research questions.

Figure 2, Figure 3 and Figure 4 summarize the current knowledge about the involvement of the peptidergic systems in the heterogeneous, complex and aggressive melanoma disease and illustrate the functional complexity involving oncogenic and anti-melanoma peptides as well as the numerous anti-melanoma strategies that have been tested to date. This review shows the enormous potential of targeting the peptidergic systems for the treatment of melanoma alone or in combination with other therapies (e.g., surgery and chemotherapy). Unfortunately, in young people there is an increased incidence of developing melanoma which represents approximately 65% of skin cancer deaths; in addition, survival dramatically decreases in melanoma stages III/IV despite recent advances in molecular-targeted drugs, molecular and genetic analysis, and proteomic, transcriptomic and genomic technologies [2,3,11,12,13,14]. This means that new research lines must be urgently investigated such as the involvement of the peptidergic systems in melanoma development, as it has been reported in other types of cancer [3]. In fact, peptidergic systems are useful in melanoma for tumor progression, diagnosis, prognosis and treatment; for metastasis development; and for increased cutaneous melanoma risk, and they also serve as a predictive factor for postoperative outcomes [50,85,87,89,92,123].

In melanoma many peptidergic systems exert oncogenic, anti-melanoma, and dual oncogenic and anti-melanoma effects, showing functional complexity but also the large number of possible research lines and therapeutic strategies currently available to combat the disease. The dual effect of peptides needs to be studied in greater depth due to the multiple factors involved (e.g., G proteins, signaling pathways, and receptor types) [24], and it must be fully understood how the same peptide acts as an oncogenic agent and how it acts as an anti-melanoma agent. A plethora of different anti-melanoma strategies have been developed including peptide/peptide receptor antibodies, peptide receptor antagonists or agonists, enzyme inhibitors, CAR-macrophages, microRNAs and vaccines [13,16,17,20,24,30,44,61,63,91,124,126,147]. In addition, many strategies for peptide delivery in melanoma favoring tumor suppression, protecting peptides from enzymatic degradation, decreasing cytotoxicity, and improving tumor selectivity have been developed [110,111].

One of the most common anti-melanoma treatments is the use of peptide receptor antagonists (valsartan, EMA401, PD-123,319, BIIE0246, SR-48,692, BQ788, A-192,621, L-733,060, L-732,138, aprepitant, ANT308) involving the angiotensin, endothelin, substance P, vasoactive intestinal peptide, α-melanocyte-stimulating hormone, neuropeptide Y and neurotensin peptidergic systems, because these peptides favor tumor progression, promoting melanoma cell proliferation, migration, invasion and metastasis as well as angiogenesis [2,44,56,63,91,124,126,140,147]. This is a crucial and promising line of research that needs to continue as well as the use of peptide receptor agonists (e.g., melanotan-II and zoladex) as anti-melanoma agents because they block all the previous oncogenic effects mentioned above [13,30,61]. Peptidergic systems open many possibilities for translational research. Peptide receptor antagonists show a higher therapeutic capacity than peptide receptor agonists/peptides because in general peptides have a poor bioavailability and a short half-life but show a high solubility and are safe. One of the most studied peptidergic systems in cancer is the substance P/neurokinin-1 receptor system and the anticancer effect mediated by the peptide receptor antagonist aprepitant via this system [159]; in fact, this anti-emetic drug has been suggested to be repurposed as a broad anticancer agent [159]. Aprepitant is safe and well tolerated [159]; therefore the substance P/neurokinin-1 receptor system and the use of aprepitant would be a good starting point to study the anti-melanoma action of this drug by increasing the dose and administration time that is currently used in clinical practice as an anti-emetic. Both previous anti-melanoma strategies could be applied in combination with standard treatments used in clinical practice or in combination with other experimental strategies (e.g., endothelin B receptor antagonists and MAPK inhibitors) [46]. Previous strategies to fight melanoma, that is the use of peptide receptor antagonists and the administration of anti-melanoma peptides/agonists, are based on the expression/overexpression of peptide receptors in melanoma cells which is crucial for diagnosis and the application of more specific and safer anti-melanoma strategies (e.g., peptide receptor agonists coupled to cytotoxic drugs) [13,126]. The current available data pave the way for conducting clinical trials in the near future testing anticancer peptides/peptide receptor antagonists as anti-melanoma agents. However, preclinical studies still need to be carried out, taking into account for example the following points: The right anticancer dose of the peptide receptor agonist/antagonist is directly associated with the size of the tumor and the total number of peptide receptors; this is important since peptide receptor occupancy close to 100% is needed for an antitumor efficacy (e.g., NK-1R antagonists) and hence the possible side-effects (e.g., diarrhea, headache, hiccups, fever, hypotension, hot flashes, insomnia, dyspepsia, dehydration, conjunctivitis, somnolence, and cognitive disorders) must be well known. It is important to know how agonists/antagonists regulate the plasma level of chemotherapeutic drugs and corticosteroids, and how agonists/antagonists influence the risk of developing chemotherapy-induced peripheral neuropathy and facilitate febrile neutropenia. All these previous points must be better defined and studied. Thus, studies regarding the highest safe dose of agonist/antagonist peptide receptors exerting the maximal antitumor effect, administration time, drug–drug interactions, tolerability, safety, efficacy, dissolution and solubility, administration route and the synthesis of new agonists/antagonists must be developed. Another interesting research line is the polymorphism of peptide receptors because it is crucial for the response of melanoma cells to peptides [75]. It is important to note that the same peptide receptor antagonist favored a plethora of anti-melanoma actions, and it is also crucial to know in melanoma all the interactions between anti-melanoma peptide receptor antagonists and anti-melanoma/oncogenic peptides. The understanding of these interactions will be of great help in knowing how the peptidergic systems regulate melanoma development and in developing more specific anti-melanoma strategies.

Peptidergic systems also regulate both melanoma microenvironment and the immune system by exerting an immunosuppressive effect and by reducing the interactions between melanoma cells and T lymphocytes, promoting the escape of melanoma cells from the immune system [45,80]. Thus, a detailed understanding of the peptidergic systems–melanoma microenvironment–immune system axis is fundamental to develop anti-melanoma strategies, as it is also important to know more details about some questions related to melanoma such as the relationships between angiotensin II and ACE [20,21,27], the renin–angiotensin system and melanoma treatment resistance/cancer stem cells [28], sex differences in the expression of the peptidergic systems in melanoma [155], chemical sympathectomy and melanoma development [125], the obesity and melanoma link [124,157], chronic infections mediated by pathogens and melanoma development [158], and methionine-enkephalin/imiquimod and opioid peptide expression [116]. It should be noted that much of the current knowledge about anti-melanoma treatments indicates a high specificity, such as the overexpression of peptide receptors in melanoma cells, compared to normal cells; CAR-macrophages exerted an anticancer activity against melanoma cells expressing a high number of endothelin B receptors but no activity was observed against melanoma cells expressing a low number of these receptors [51], and TRH favored the proliferation of melanoma cells, but this was not observed in melanocytes [145].

In sum, a meticulous and in-depth study of the peptidergic systems will help to understand how peptidergic systems regulate melanoma progression and shed light on possible therapeutic applications that could be used in clinical practice in the near future. This review shows the enormous anti-melanoma potential of the peptidergic systems.

## Figures and Tables

**Table 1:** Oncogenic peptides in melanoma.

Peptides	Oncogenic Effects
Adrenomedullin	Promoted melanoma cell growth, migration, invasion and angiogenesis [16] Melanoma cells expressed adrenomedullin and its receptors [16] Hypoxia favored adrenomedullin expression [16]
Angiotensin	ACE overexpressed in melanoma cells compared with melanocytes [20] Angiotensin II, via ACE, promoted melanoma cell proliferation and migration [20] Angiotensin II/Y6AII favored human melanoma cell proliferation [24] Angiotensin II promoted melanoma lung metastasis [23] Lisinopril, EMA401 and losartan promoted human MV3 melanoma cell migration and invasion [21] Losartan/PD-123,319 favored melanoma cell adhesion and invasion [22]
Bradykinin	Favored melanoma cell migration and invasion [33] Melanoma cells expressed kinin B_2_ receptors [34]
Corticotropin-Releasing Hormone	CRH increased melanoma cell migration [39] CRH–pro-opiomelanocortin axis related to malignant melanomas [37] Melanoma metastasis: lower CRH expression in men than in women; a higher CRH expression associated with decreased overall survival in men [36]
Endothelin	Endothelin-1 contributes to melanoma cell proliferation, migration and invasion [42] Tumor microenvironment endothelin-1 expression increased with advancing stages of melanocyte transformation [55] Melanoma cells overexpressed endothelin-1 [53] Endothelin-3 overexpression exerted an immunosuppressive effect in melanoma microenvironment [45]
Hemokinin-1	Hemokinin-1 increased melanoma cell migration [63]
α-Melanocyte-Stimulating Hormone	α-melanocyte-stimulating-hormone-induced melanoma cell proliferation [104] α-melanocyte-stimulating hormone expression and plasma level augmented in patients with melanoma [40,86] Human primary cutaneous melanoma showed a higher expression of α-melanocyte-stimulating hormone than that observed in melanocytic nevi; no expression was reported in melanoma metastases [85] α-melanocyte-stimulating hormone high expression in malignant melanomas [37] α-melanocyte-stimulating hormone reduced interaction between T lymphocytes and melanoma cells favoring melanoma cell escape from the immune system [57] Melanocortin 1 receptor activation in melanoma cells impaired tumor T cell infiltration reducing anticancer immunity [90] Higher expression of melanocortin 1 receptors related to shorter survival in metastatic and primary melanomas and poor prognosis [87,90] Patients with melanoma showing a low expression of melanocortin 1 receptors had a better prognosis than those expressing a high level [89] Melanocortin 1 receptor variants increased cutaneous melanoma risk [92]
Oxytocin	Oxytocin receptor mediated angiogenesis, lung metastasis and melanoma cell migration and invasion, but not cell proliferation [127] Oxytocin receptor upregulation in malignant melanomas [127] Chronic restraint stress increased oxytocin plasma levels, favored melanoma cell lung metastasis and decreased survival [127]
Substance P	Substance P promoted melanoma cell proliferation [140] Substance P expressed in metastatic melanomas, primary invasive malignant melanomas, melanomas in situ, spindle and epithelioid cell (Spitz) nevi and atypical nevi, but not in benign melanocytic nevi [135] Neurokinin-1 receptor/*TACR1* gene overexpressed in melanoma cells [142] Melanoma cells and human samples expressed neurokinin-1 receptors [141] Melanoma cell lines expressed mRNA for the neurokinin-1 receptor [141] Neurokinin-1 receptor involved in melanoma cell viability [141]
Thyrotropin-Releasing Hormone	TRH favored the proliferation of melanoma cells, but this was not observed in melanocytes [145] TRH detected in melanoma cell lines [145] TRH immunoreactivity in benign nevi, dysplastic nevi and melanomas; expression higher in dysplastic nevi than in benign nevi: predictive tool for melanoma development [145] Thyrotropin-releasing hormone binds to human melanocortin 1 receptor [144]

**Table 2:** Anti-melanoma peptides.

Peptides	Anticancer Effects
Angiotensin	Angiotensin II type 2 receptor activation inhibited melanoma cell and transendothelial migration and metastasis [25] Ectopic *AGTR1* expression in melanoma cell lines blocked cell proliferation [24] Losartan inhibited melanoma cell migration [22] EMA401/PD-123,319 blocked angiogenesis and melanoma growth [24] Melanoma cell proliferation inhibited with ACE silencing or ACE inhibitors (lisinopril) [20]
Bradykinin	Kinin B_1_ receptor activation counteracted melanoma tumor growth and metastasis [30,31] des-Arg^9^-bradykinin (DABK) decreased melanoma metastasis and increased survival [30,32]
Calcitonin Gene-Related Peptide	Promoted apoptosis in melanoma cells, increased expression levels of total/cleaved caspases 3/9 and increased Bax/Bcl-2 ratio [35]
Corticotropin-Releasing Hormone	CRH decreased melanoma cell proliferation [36] CRH expression in squamous cell carcinomas, basal cell carcinomas, melanocytic nevi and melanomas [36]
β-Endorphin	Melanoma cells, producing β-endorphin, reduced tumor growth and increased immune cell infiltration [41] β-Endorphin expression: higher in advanced/metastatic melanomas than in benign melanocytic nevi [3,40] Positive association between tumor progression and β-endorphin expression in human melanoma tissues [41]
Endothelin	Fentanyl citrate decreased the release of endothelin-1 (oncogenic) mediated by bradykinin in melanoma cells [34] Endothelin B receptor/*EDNRB*-mediated melanoma suppressive action [52] Endothelin-3 favored survival in metastatic melanoma [54] Endothelin-3 gene sequence-specific shRNA vector pLVTHM-endothelin-3-RNAi transfected into melanoma cells: decreased melanoma cell proliferation, inhibited tumor growth, cell migration and invasion, and increased apoptosis [54] Endothelin-3 expressed in melanoma cells and metastatic melanoma [54]
Gonadotropin-Releasing Hormone	Gonadotropin-releasing hormone agonists decreased melanoma cell proliferation, migration, invasion, metastasis and angiogenesis [61,62] Melanoma cells expressed gonadotropin-releasing hormone receptors [61]
Kisspeptin	Metastasis-suppressor gene KISS1 upregulation blocked melanoma cell proliferation and migration [65] KISS1 and Let-7i downregulated in patients with melanoma [65] KISS1 inhibitors favored melanoma cell proliferation and migration which were counteracted with Let-7i [65] Kisspeptin 54 increased vemurafenib pro-apoptotic effect in vemurafenib-resistant melanoma cells [67] Metastatic melanomas: kisspeptin mRNA expression downregulation [66] Kisspeptin hypothalamic expression was lower in healthy animals than in those with melanoma: it seems that tumors increased the synthesis of hypothalamic kisspeptin which exerts antiproliferative and antimetastatic effects [64]
α-Melanocyte-Stimulating Hormone	α-melanocyte-stimulating hormone blocked invasive and metastatic capacities of melanoma cells [73] α-melanocyte-stimulating hormone blocked melanoma cell migration and decreased uveal melanoma cell invasion [74,82] α-melanocyte-stimulating hormone exerted anti-invasive and anti-inflammatory effects in melanoma cells expressing the wild-type melanocortin 1 receptor [75] α-melanocyte-stimulating hormone/melanocortin 1 receptor system decreased melanoma risk development by maintaining melanocytes genomic stability [71]
Melittin	Melittin blocked melanoma cell growth, migration, and invasion, promoted apoptosis and increased survival [106,108] Melittin–mertansine promoted apoptosis in M2 macrophages, inhibited melanoma cell growth, migration and invasion and improved survival rate [1] Melittin–dKLA inhibited M2 macrophage proliferation and migration leading to melanoma growth decrease [109]
Methionine-Enkephalin	Methionine-enkephalin decreased melanoma cell growth, tumor volume and increased survival and opioid receptor expression [115,116,117] Methionine-enkephalin level reduced in melanocytic tumors [114]
Neuropeptide Y	Thinner melanoma tumors related with a higher neuropeptide Y expression [123] Low neuropeptide Y expression associated with high melanoma cell proliferation [123] High neuropeptide Y expression: better prognostic and outcome [123] Neuropeptide Y expressed in primary cutaneous melanoma and melanocytic nevi but not in melanoma metastasis [85]
Somatostatin	Somatostatin analogs blocked uveal melanoma cell proliferation [131] Somatostatin receptors in melanoma/uveal melanoma cell lines and samples [128,129,130,132]
Substance P	Substance P promoted apoptosis in melanoma cells [139] Pretreatment with substance P prevented/delayed tumor development and favored the action of immune mediators exerting a protective effect against melanoma [136] Substance P blocked melanoma growth and potentiated the inhibitory action mediated by radiotherapy [138]
Caerin Peptides	Caerin peptides blocked melanoma cell proliferation and downregulated lipid metabolites [154] Caerin peptides increased the level of 3-hydroxyvalproic acid and carnitine derivatives involved in anti-inflammatory and antiproliferative effects [154]
Cationic Peptides	Cationic peptides exerted cytotoxic effects against melanoma cells [151] Peptide PEPAD decreased cell migration and promoted apoptosis in melanoma cells [152]
IK14004	IK14004 inhibited lung melanoma progression without compromising immune tolerance [14]
KW18	KW18 promoted apoptosis in melanoma cells: safe therapeutic agent for drug-resistant melanoma treatments [150]
^D^PMI-ω	^D^PMI-ω blocked melanoma cell growth [153] ^D^PMI-ω and anti-PD-1 antibodies co-administration: increased immunotherapy efficacy [153]
pYEEIE-RELNs-DOX	pYEEIE-RELNs-DOX inhibited melanoma growth, and no toxicity was observed in kidneys, lungs, spleen, liver and heart [155]
ZCPAN	ZCPAN promoted oxidative injury and apoptosis in melanoma cells [10]

**Table 3:** Peptides and anti-melanoma treatments.

Peptides	Anti-Melanoma Strategies
Adrenomedullin	Anti-adrenomedullin or anti-adrenomedullin receptor antibodies: reduced melanoma cell growth, migration, invasion, angiogenesis and lymphangiogenesis [16] mRNA vaccines: decreased angiogenesis and size/number of lung metastases and increased the number of CD8^+^ T cells [17]
Angiotensin	Angiotensin I-converting enzyme silencing/inhibitors (Lisinopril): blocked melanoma cell proliferation [20] Angiotensin II type 1 receptor antagonist (valsartan): inhibited oncogenic effects mediated by angiotensin II [23] Co-administration of anti-programmed death-1 antibody and valsartan: high anti-melanoma growth action [26] Angiotensin II type 2 receptor antagonists (EMA401/PD-123,319): inhibited melanoma growth/angiogenesis and potentiated MEK/BRAF inhibitors in cells with V600 mutations [120] Anti-E-selectin antibodies: inhibited lung metastases induced by angiotensin II [23]
Bradykinin	Kinin B_1_ receptor activation/kinin B_1_ receptor agonists: counteracted melanoma tumor growth/metastasis and increased survival [30]
Corticotropin-Releasing Hormone	PD-098059 (ERK1/2 blocker): decreased melanoma cell migration [39]
Endothelin	Endothelin B receptor antagonists: reduced the number of lymphatic/blood vessels and melanoma growth [44] Endothelin/endothelin B receptor system and MAPK inhibition: decreased tumor growth and increased survival [46] MAPK blockade: increased anti-endothelin B receptor drug conjugates efficacy by favoring target expression in melanoma [47] Rendomab B4 antibody (directed against endothelin B receptors): blocked melanoma cell migration [48] shRNA molecules against endoglin: anti-angiogenic actions in endothelial cells and anticancer effects in melanoma cells [53] Endothelin-3 silencing: counteracted malignant melanoma cell behavior [54]
Gastrin-Releasing Peptide	Vaccines (anti-mGM-CSF/mGGn/anti-mGM-CSF/GRP6): inhibited melanoma by decreasing tumor volume and weight [60]
Gonadotropin-Releasing Hormone	Gonadotropin-releasing hormone receptor agonists (zoladex, goserelin acetate): inhibited melanoma cell migration and invasion and angiogenesis [61,62]
Hemokinin-1	Neurokinin-1 receptor antagonist (L-732,138): inhibited melanoma cell migration [63]
Kisspeptin	Let-7i (microRNA) upregulation: counteracted melanoma cell proliferation and migration and promoted apoptosis [65]
α-Melanocyte-Stimulating Hormone	Beta (1)-integrin subunit antibody: reduced melanoma cell migration [74] Melanotan-II (melanocortin 1 receptor agonist) coupled to camptothecin (cytotoxic drugs): reduced melanoma cell growth [13] ML00253764 (melanocortin 4 receptor antagonist) alone or in combination with vemurafenib (B-rafV600E inhibitor): exerted pro-apoptotic and antiproliferative effects [91] *Pro-opiomelanocortin* gene delivery: blocked melanoma growth and metastasis by attenuating adhesive and migratory capacities [84] 8-Methoxybutin (microphthalmia-associated transcription factor inhibitor): blocked α-melanocyte-stimulating hormone-induced melanoma cell proliferation [104]
Melittin	Temozolomide (chemotherapeutic drug) and melittin: more effective at inhibiting melanoma cell growth and invasion, compared to melittin or temozolomide administered alone [106] Diallyl trisulfide (DATS): promoted apoptosis in melanoma cells [107] Hepatitis B core virus-like particles (HBc VLPs): improved tumor selectivity, decreased cytotoxicity, protected melittin from enzymatic degradation and favored tumor suppression [110] RGD (Arg-Gly-Asp)-melittin: promoted apoptosis, blocked melanoma cell proliferation, migration and invasion and inhibited chemotaxis [111] Hypochlorous-acid-treated melanoma cells: inhibited tumor growth, promoted cytotoxic T lymphocyte infiltration, increased anticancer effects of immune checkpoint blockade, and augmented survival [112] Melittin-RADA_32_-CpG-lysate vaccine: killed melanoma cells, activated dendritic cells and favored cytotoxic T lymphocytes tumor microenvironment infiltration [113]
Methionine-Enkephalin	Imiquimod: upregulated the opioid growth factor receptor facilitating the anticancer action mediated by methionine-enkephalin [118] Imiquimod topical administration: good results, well tolerated and safe for melanoma cutaneous metastasis [3,122]
Neuropeptide Y	Neuropeptide Y_2_ receptor antagonists (BIIE0246): blocked melanoma growth by targeting angiogenesis processes [124] Chemical sympathectomy (6-hydroxydopamine hydrobromide): decreased melanoma tumor weight [125]
Neurotensin	Neurotensin 1 receptor antagonists (SR-48,692): promoted melanoma cell cycle arrest and apoptosis [126]
Oxytocin	Knocking down β-arrestin 2 or the oxytocin receptor: counteracted lung metastasis of melanoma cells and increased survival [127]
Somatostatin	Paclitaxel formulation of solid lipid nanoparticles modified with Tyr-3-octreotide: promoted apoptosis and reduced invasion in melanoma cells, decreased tumor volume, favored systemic immune response and decreased nodule formation number in lung metastasis experimental models [133]
Substance P	Neurokinin-1 receptor antagonists (aprepitant, L-733,060, L-732,138): favored apoptosis in melanoma cells and blocked substance P mitogen stimulation of melanoma cells [2,140] Cyclosporin A (immunosuppressive agent): blocked melanoma cell growth and promoted apoptosis [142]
Vasoactive Intestinal Peptide	Vasoactive intestinal receptor antagonists (ANT308): inhibited melanoma cell proliferation and migration, promoted apoptosis, decreased N-cadherin/melanoma cell adhesion molecule expressions, and reduced tumor volume and the number/size of liver metastases [147] VPAC2 receptor knockdown: blocked melanoma cell proliferation and migration [147]

## Data Availability

No new data were created or analyzed in this study. Data sharing is not applicable to this article.
